# WGS-Based Lineage and Antimicrobial Resistance Pattern of *Salmonella* Typhimurium Isolated during 2000–2017 in Peru

**DOI:** 10.3390/antibiotics11091170

**Published:** 2022-08-30

**Authors:** Raquel Hurtado, Debmalya Barh, Bart C. Weimer, Marcus Vinicius Canário Viana, Rodrigo Profeta, Thiago Jesus Sousa, Flávia Figueira Aburjaile, Willi Quino, Renan Pedra Souza, Orson Mestanza, Ronnie G. Gavilán, Vasco Azevedo

**Affiliations:** 1 Laboratório de Genética Celular e Molecular, Departamento de Genética, Ecologia e Evolução, Instituto de Ciências Biológicas, Universidade Federal de Minas Gerais, Belo Horizonte 31270-901, Brazil; 2 Institute of Integrative Omics and Applied Biotechnology (IIOAB), Nonakuri, Purba Medinipur 721172, West Bengal, India; 3 Department of Population Health and Reproduction, School of Veterinary Medicine, 100K Pathogen Genome Project, University of California, Davis, CA 95616, USA; 4 Veterinary Medicine Department, Veterinary School, Universidade Federal de Minas Gerais, Belo Horizonte 31270-901, Brazil; 5 Instituto Nacional de Salud, Lima 15072, Peru; 6 Laboratório de Biologia Integrativa, Departamento de Genética, Ecologia e Evolução, Instituto de Ciências Biológicas, Universidade Federal de Minas Gerais, Belo Horizonte 31270-901, Brazil; 7 Escuela Profesional de Medicina Humana, Universidad Privada San Juan Bautista, Lima 15072, Peru

**Keywords:** antimicrobial resistance, multi-drug resistance, *Salmonella* Typhimurium, whole-genome sequencing, resistance plasmids, antimicrobial susceptibility test, GWAS

## Abstract

*Salmonella* Typhimurium is associated with foodborne diseases worldwide, including in Peru, and its emerging antibiotic resistance (AMR) is now a global public health problem. Therefore, country-specific monitoring of the AMR emergence is vital to control this pathogen, and in these aspects, whole genome sequence (WGS)—based approaches are better than gene-based analyses. Here, we performed the antimicrobial susceptibility test for ten widely used antibiotics and WGS-based various analyses of 90 *S.* Typhimurium isolates (human, animal, and environment) from 14 cities of Peru isolated from 2000 to 2017 to understand the lineage and antimicrobial resistance pattern of this pathogen in Peru. Our results suggest that the Peruvian isolates are of Typhimurium serovar and predominantly belong to sequence type ST19. Genomic diversity analyses indicate an open pan-genome, and at least ten lineages are circulating in Peru. A total of 48.8% and 31.0% of isolates are phenotypically and genotypically resistant to at least one antibiotic, while 12.0% are multi-drug resistant (MDR). Genotype–phenotype correlations for ten tested drugs show >80% accuracy, and >90% specificity. Sensitivity above 90% was only achieved for ciprofloxacin and ceftazidime. Two lineages exhibit the majority of the MDR isolates. A total of 63 different AMR genes are detected, of which 30 are found in 17 different plasmids. Transmissible plasmids such as lncI-gamma/k, IncI1-I(Alpha), Col(pHAD28), IncFIB, IncHI2, and lncI2 that carry AMR genes associated with third-generation antibiotics are also identified. Finally, three new non-synonymous single nucleotide variations (SNVs) for nalidixic acid and eight new SNVs for nitrofurantoin resistance are predicted using genome-wide association studies, comparative genomics, and functional annotation. Our analysis provides for the first time the WGS-based details of the circulating *S.* Typhimurium lineages and their antimicrobial resistance pattern in Peru.

## 1. Introduction

*Salmonella* Typhimurium, and other non-typhoidal *Salmonella* (NTS), are responsible for foodborne illnesses worldwide [1]. NTS can cause gastrointestinal disease, progressing to systemic infections in some patients. The World Health Organization (WHO) recently declared *Salmonella* a high-priority pathogen due to increased resistance to first-line antibiotics, fluoroquinolones, and third-generation cephalosporins [2]. In Peru, 234 foodborne outbreaks were reported between 2014 and 2018. In 2019, 1,204,136 diarrheal cases were reported mainly due to contaminated drinking water and food [3,4]. *S. enterica* serovars, mainly Infantis, Enteritidis, and Typhimurium, were associated with gastroenteritis in Lima hospitals during 2008–2013 and 2015–2017 [5,6]. Additionally, multi-drug resistant (MDR) *Salmonella* Infantis isolates resistant to first-line antibiotics, third-generation cephalosporins, and ciprofloxacin antibiotics are highly prevalent in Peru [7,8]. Unfortunately, limited studies are using whole-genome sequencing (WGS) strategies to understand diversity, alignment with antimicrobial resistance (AMR) phenotype and the AMR prevalence of circulating *Salmonella*.

*S.* Typhimurium has a broad host range and emerging dominant MDR phenotypes. For example, the MDR DT104 group disseminated rapidly globally [9], and ST313, an MDR group, is responsible for invasive diseases in Africa [10]. Apart from chromosomal mobile AMR genes mobile genetic elements, such as plasmids and pathogenic islands, are essential in expanding AMR distribution among the population and are often associated with hospital-acquired infections and foodborne outbreaks [8,9,10,11,12]. MDR *Salmonella* isolated in various countries largely contain genes for β-lactam, tetracycline, aminoglycoside, and quinolone resistance on plasmids [13,14]. 

The use of WGS in molecular epidemiology and AMR surveillance has several advantages as compared to conventional PCR, other molecular methods, or phenotypic approaches [15,16,17]. Since *S*. Typhimurium exhibits a broad and diverse host range, pathogenicity, and risk to human health [18,19,20]; WGS-based comparative and phylogenetic analysis as part of AMR surveillance is a promising approach to rapidly predict resistances that is much faster than phenotypic methods. Reports indicate that WGS-based approaches to predicting antimicrobial susceptibility with a good correlation between genotype and phenotype, detecting and tracing outbreaks, and determining the complement of AMR determinants are essential resources in the appropriate selection of antibiotic treatment in *S.* Typhimurium infections [8,21,22,23,24]. Furthermore, this approach allows the discovery of new AMR genes, or alleles of known AMR genes, as reported in various pathogenic bacteria [12,25,26]. Comparative genomics and genome-wide association studies (GWAS) have identified these potential causal variants associated with virulence and with AMR in multiple organisms [26,27].

**Figure 1:**
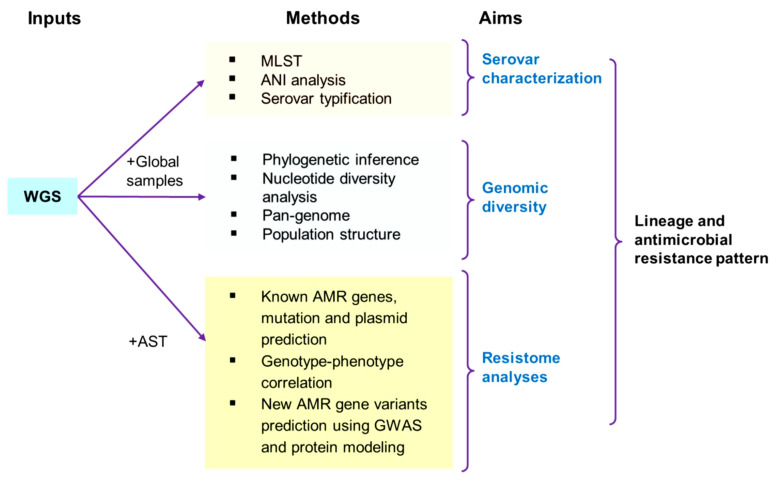
The overview of methods and objectives of this study.

In this study, we used whole genome sequences of *S.* Typhimurium isolates from a Peruvian surveillance study to determine its Peru-specific lineage and antimicrobial resistance pattern. More specifically, we classified the *S.* Typhimurium serovars using serotyping, multi-locus sequence typing, and average nucleotide identity (ANI) analysis approaches. Further, the nucleotide diversity, phylogenetic, pan-genome, and population structure analyses were carried out to determine the genome diversity of the circulating Peruvian *S.* Typhimurium isolates. Finally, identification and characterisation of AMR genes, GWAS-based prediction of new non-synonymous single nucleotide variation (SNVs) for AMR, prediction of AMR effect of newly identified SNVs, and genotype–phenotype correlations were performed for antimicrobial resistome profiling of these isolates. The overview of the methods applied, and the objectives of this study are represented in Figure 1. 

## 2. Materials and Methods

### 2.1. Samples and Collection Sites

We examined 90 pathogenic *S.* Typhimurium isolated from humans (*n* = 78), animals (*n* = 3) and the environment (*n* = 9). The isolates were collected between 2000 and 2017 from 14 cities in Peru, as shown in the global map (https://glenjasper.github.io/leaflet-salmonella). 

### 2.2. Antibiotic Susceptibility Test 

The disk diffusion method [28] was used to determine AMR following Committee for Clinical Laboratory Standards (CLSI) guidelines for ten commonly used antibiotics: ampicillin (AM), chloramphenicol (C), ciprofloxacin (CIP), trimethoprim-sulfamethoxazole (SXT), cefotaxime (CTX), nalidixic acid (NA), amoxicillin-clavulanate (AMC), nitrofurantoin (N), tetracycline (TE), and ceftazidime (CAZ). MDR was defined as resistance to at least three antibiotics. The intermediate or reduced susceptibility phenotype was considered a susceptible isolate (Supplementary Table S1). 

### 2.3. Whole Genome Sequencing

We used Peruvian *Salmonella* Typhimurium genome (90 isolates) sequence raw reads generated under the 10K *Salmonella* Project (BioProject: PRJEB35182). After assembly and annotation, we submitted these genomes to the BioProject (PRJNA635403) as “Peruvian *Salmonella* spp. Genome sequencing and assembly”. The genome sequencing was performed within the 10K *Salmonella* Project as described by Perez-Sepulveda et al., 2021 [29]. Additionally, a global set of 50 genomes were obtained from the Genbank database and included in the further analysis to estimate the diversity and ancestry of Peruvian isolates globally (Supplementary Table S2).

### 2.4. Genome Assembly, Annotation, and Plasmid Detection

The raw fastq sequences from the Illumina 150 bp paired-end were checked for quality using FastQC v0.11.8 [30]. De novo genome assembly was performed using Unicycler v0.4.5 [31], and the quality of the assembly was evaluated using Quast v3.2 [32] and BUSCO v4.0.6 [33]. Contigs < 200 bp were removed, and the assembled genomes were submitted under BioProject: PRJNA635403. For further analysis, the annotation was performed using PROKKA v1.11 [34], and only the genome sequences with >20× depth coverage were considered. We considered 20× to be the minimum coverage to include the maximum number of our isolates (*n* = 90 out of a total *n* = 109) for core genome analysis, variant determination, and monitoring of infectious outbreaks in other previous studies [35,36,37]. Plasmids were predicted and reconstructed from the assembled genomes using MOB-suite [38] and classified as conjugative, mobilisable, and non-mobilisable plasmids. PlasmidFinder [39] and ABRicate (https://github.com/tseemann/abricate; accessed on 4 April 2022) were also used to crosscheck the MOB-suite results.

### 2.5. In Silico Serotyping, MLST, and ANI Analysis

The serovar and sequence types were predicted using SeqSero [40] and MLST (https://github.com/tseemann/mlst; accessed on 5 April 2022) [41], respectively. Pairwise average nucleotide identity (ANI) values were calculated using FastANI v.1.1 [42] to determine the degree of genomic relatedness. Results were visualised using the ggplot2 V.3.3.5 (https://ggplot2.tidyverse.org/reference/index.html; accessed on 5 April 2022). The respective tools’ assembled genomes and default parameters were used for these analyses.

### 2.6. Pan-Genome and Phylogenetic Analysis

Core and accessory genes were identified using Roary v3.12 [43] with default settings. The R package micropan v.2.1 [44] was used to model the openness of the pan-genome using Heaps’ law as described by Tettelin et al., [45] with the number of permutations set to 1000. Values α ≤ 1 representant an open pan-genome, where adding new genomes will increase the pan-genome substantially. For this analysis, we used our Peruvian samples and 50 *S.* Typhimurium isolates from 29 countries distributed on six continents (Asia, Europe, Africa, Australia, and North and South America). We used *S.* Typhimurium (LT2, GenBank: NC_003197) as the reference. Core genome-SNPs were predicted using Snp-sites [46] from the Roary v3.12 [43] core genome output. Phylogenetic analysis was based on core genome genes of the Peruvian and 140 global samples. The RaxML v8.2.12 [47], maximum likelihood method, GTR + Gamma model, and 1000 bootstrap replicates were applied to create the Phylogenetic trees that were visualised using the ggtree package in R (R Development Core Team, 2016).

### 2.7. Population Structure and Diversity Analyses

The population structure for two sample sets was determined separately using core gene SNPs: (i) Peruvian and (ii) Peruvian + 50 global samples. The Bayesian analysis of population structure (BAPS) [48] model was defined using RhierBAPS v1.0.1 [49]. The nucleotide variation analysis within the Peruvian and global *S.* Typhimurium populations was calculated using the pairwise similarity (inverse diversity calculation) and median pairwise similarities using the core genome SNPs and the MEGA-X tool [50]. Ggplot2 package in R (R Development Core Team, 2016) was used to visualise the results. These analyses allow estimating the diversity and ancestry of Peruvian isolates from global representative isolates.

### 2.8. Identification of Known Antimicrobial Resistance Genes and Single Nucleotide Variations

For each genome, the AMR-associated genes were identified in the chromosome and plasmids using ABRicate (https://github.com/tseemann/abricate; accessed on 4 April 2022) using CARD [51] and ARG-ANNOT [52] databases. More than 85.0% of sequence coverage and identity were considered the lower limit. Additionally, AMR Finder [53] and ResFinder [54], contain a large number of *Salmonella* spp. sequences, and were included to corroborate the results. The AMR genes were classified according to resistance mechanisms and drug class using the CARD database and manual curation [51].

To identify the SNPs in the AMR genes from the chromosomal DNA, first, we extracted the AMR gene sequences using Roary [43], followed by analysis with MAFFT v7.307 [55] for sequence alignment. Finally, SNP-sites [46] was used for the variant calling. Subsequently, we used PointFinder [56] to identify alleles and associate them with AMR.

### 2.9. Genotype–Phenotype Correlation

We performed the genotype–phenotype correlation (GPC) analysis using disc diffusion antimicrobial susceptibility test results in combination with the WGS-informed AMR analysis. These assays were compared with the occurrence of known AMR genes and alleles associated with the resistance to the respective drug. False-positive, false-negative, sensitivity, specificity, and accuracy of the GPC were calculated as described previously [21]. Finally, phenotypically resistant isolates that did not contain known genes for this resistance were identified as candidates for use in finding new variants that may confer resistance.

### 2.10. Genome-Wide Association Study to Identify New Single Nucleotide Variations Associated with Resistance Phenotype

In isolates where genotype–phenotype association did not match, we used the genome-wide association study (GWAS) approach to identify possible genes and alleles for specific AMR. A similar approach, as described by Bandoy and Weimer [57], was used for the GWAS analysis with a chi-square test in R (R Development Core Team, 2016) to identify mutant alleles that conferred a specific phenotype. In this process, the variant calling was performed for all the isolates using LT2 isolate as the reference. Only the phenotype observed for the ten tested drugs was considered. The Snippy v3.2 (https://github.com/tseemann/snippy; accessed on 16 April 2022) was used for variant calling, and the SNPs and indels were filtered considering a minimum sequencing depth > 20x and minor allele frequency less than 0.02 were removed. A significant association was considered when *p* < 0.05, and the values were automatically corrected for multiple testing using the Bonferroni method [58]. The results were visualised with the Manhattan plot in R (R Development Core Team, 2016) using the “qqman” package. To minimise the false positive association in the GWAS analysis, we applied the population structure as a covariable in Firth’s logistic regression analysis using the “logistf” v1.24 package in R (https://github.com/vicbp1/Genetic-Arquitecture-of-Zika; accessed on 16 April 2022). The population structure was predicted using principal component analysis (PCA) and PLINK v1.90b6.9 [59], considering the first six principal components (PC1–PC6) as continuous covariables.

### 2.11. Association of New Single Nucleotide Variations and Drug Resistance

The GWAS represent a powerful approach to identifying new genetic variants in isolates that demonstrated phenotypic drug resistance but did not contain determinants associated with known antimicrobials. We examined the potential for additional gene variants that may explain the specific drug resistance in those isolates. When found, we adopted the strategy described by Ferla et al. [57,60]. Additionally, we modelled the 3D structure of the protein harbouring the variation using the corresponding amino acid sequence and Swiss Model homology server [61]. The 3D structure was then used to predict the stability of these proteins for the new variations using the Dynamute2 tool [62]. Finally, the stability/instability property of the new allele was correlated with the observed drug resistance phenotype.

## 3. Results

### 3.1. Genomic Characterisation Shows Peruvian Salmonella Samples Belong to Typhimurium Serovar and Sequence Type ST19

Ninety WGS samples passed the quality metrics and were used for further analysis (Supplementary Table S1). The SeqSero and MLST analysis confirmed that 83 belong to serovar Typhimurium and 85 belong to sequence type ST19, respectively (Supplementary Table S1). For the global samples, the majority were serovar Typhimurium and sequence type ST19. Use of ANI analysis found the Peruvian genomes were >99.6% identical, while the global samples (*n* = 140) were >99.4% identical (Supplementary Figure S1). These analyses confirm that both samples belong to serovar S. Typhimurium and use all the isolates in subsequent analyses.

### 3.2. Pan-Genome Structure and Nucleotide Diversity of Peruvian Samples

**Figure 2:**
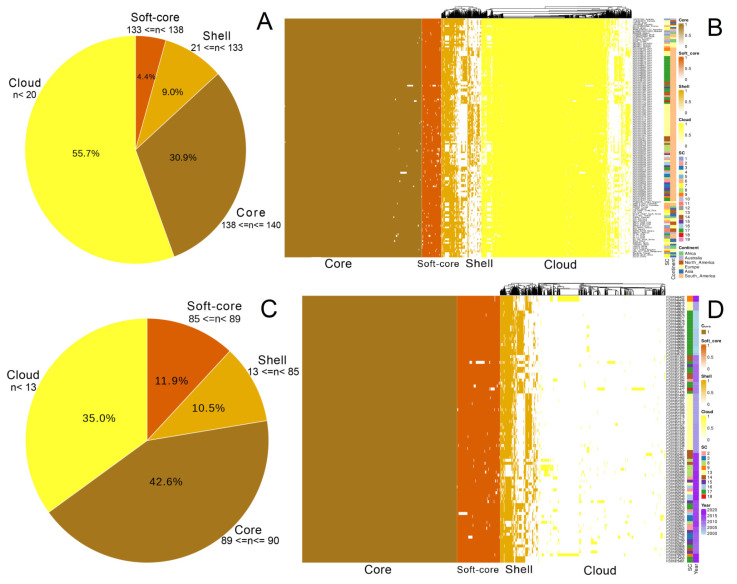
Pan-genome analyses of *S.* Typhimurium isolates. (**A**,**C**), Pie charts showing the proportion of repertoire genes in the core, soft-core, shell, and cloud of the pangenome of the global (*n* = 140) and Peruvian *S.* Typhimurium isolates (*n* = 90). (**B**,**D**), Gene presence-absence matrix shows the gene distribution in each genome. Heatmap legends on the right of panels (**B**,**D**) indicate the cluster sequence to which each sample belongs. The accessory genome of 69.1% and 57.4% presents a high diversity of gene content distinguished by sequence clusters (lineages) for Peruvian and global samples.

The pan-genome analysis examined the content of gene diversity, and the core genome was used as input data to analyse the phylogeny, population structure, and nucleotide variation within these isolates. The pan-genome of 140 *S*. Typhimurium was open (α = 0.53) and contained 11,168 orthologous genes with 3455 core genes and 7713 accessory genes (softcore = 495, shell = 1000, and cloud = 6218) that represent the 30.9% and 69.1%, respectively (Figure 2A,B). Specifically for the Peruvian isolates (*n* = 90), the core and accessory genome constitutes 42.6% and 57.4%, respectively, where there are 7462 orthologous genes, 3181 core genes and 4281 accessory genes (softcore = 889, shell = 781, and cloud = 2611) (Figure 2C,D). We identified values > 85.0% and 70.0% of pairwise nucleotides dissimilarity (Supplementary Figure S2) between the overall and within-population nucleotide diversity to be 0.057 and 0.135 for global and Peruvian samples, respectively, indicating that our Peruvian isolates are more genetically diverse and present an open pan-genome with α value minor to 1 (α = 0.73).

### 3.3. Ten Lineages of S. Typhimurium Classified under Two Major Clades Are Circulating in Peru

**Figure 3:**
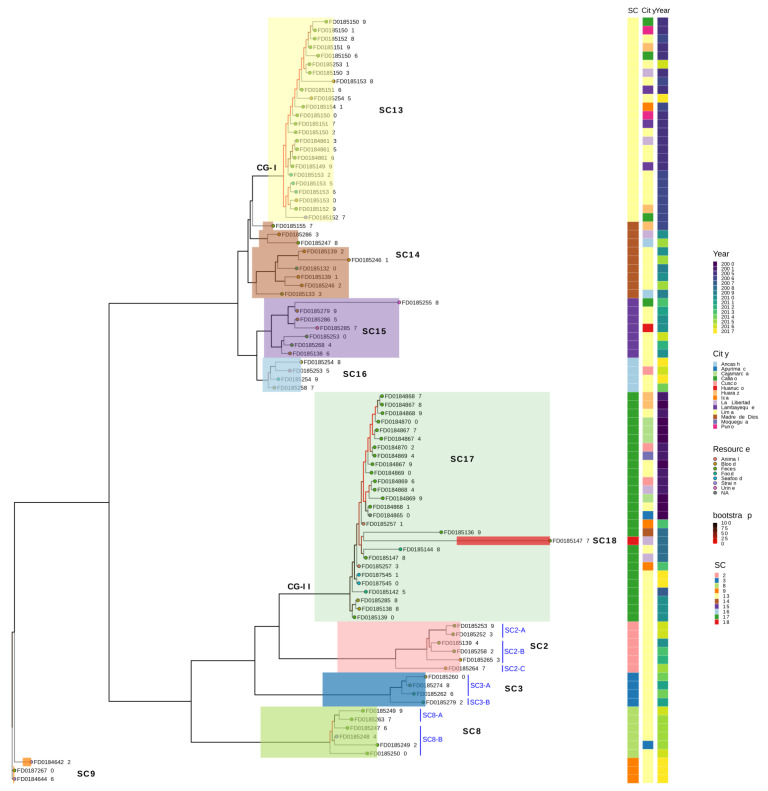
The phylogenetic tree was generated using a maximum likelihood method with 1000 replicates of bootstrap using GTR + GAMMA to estimate the evolutionary distance between Peruvian isolates (*n* = 90). The phylogenetic tree was clustered into at least two large clades and separated into nine sub-clades. Each sub-clade corresponds to a population group, except for SC18. Two emerging clades (CG-I and CG-II) are also found. Additionally, we identified subgroups based on the prediction of only the Peruvian population structure, but they did not show the phylogenetic distinction.

The un-rooted phylogenetic tree based on the core genome clustered similarly for both the global and Peruvian samples. The Peruvian isolates were analysed with the other global samples and contained ten distinct sub-clades (Supplementary Figure S3). It is also observed that the lineages of Peruvian isolates are distributed across the continents (Figure 3). When analysed exclusively for the 90 Peruvian isolates, the phylogenetic tree shows a similar number of sub-clades (Figure 3). Both analyses also found that the Peruvian samples are mostly distributed in two major clades. When we determined the population structure as sequence clusters (SC, assigned as lineages) by a two-level hierarchical Bayesian approach (BAPS) using the core gene SNPs, we observed at least 19 sequence clusters for the global samples and 10 sequence clusters for the 90 Peruvian samples within this global sample pool (Supplementary Figure S3). The first clade (*n* = 47) includes the sequences cluster SC13 (*n* = 24), SC14 (*n* = 9), SC15 (*n* = 7), SC16 (*n* = 4), and SC9 (*n* = 3); while the second clade (*n* = 43) includes SC2 (*n* = 6), SC3 (*n* = 4), SC8 (*n* = 6), SC17 (*n* = 26), and SC18 (*n* = 1) (Figure 3).

While we further analysed the sequence clusters only for the 90 Peruvian samples, we identified four additional sub-populations without much phylogenetic difference, where SC2 consists of three sub-sequence clusters and SC3 and SC8 had two sub-sequence clusters each (Figure 3). Therefore, ten sequence clusters or lineages are circulating in Peru. The major clonal group CG-I (SC3) consists of 24 isolates, distributed in seven different Peruvian cities, mostly isolated during 2005–2006, and closely related to a Switzerland sample. The second crucial clonal group, CG-II (SC17), consists of 26 isolates, is found in eight different Peruvian cities, isolated during 2000–2001, and is closely related to a Chilean sample (Figure 3).

### 3.4. The Phenotypic Profile Shows 48.9% Resistance to at least One Drug in Isolates Circulating in Peru for Ten Drugs

**Figure 4:**
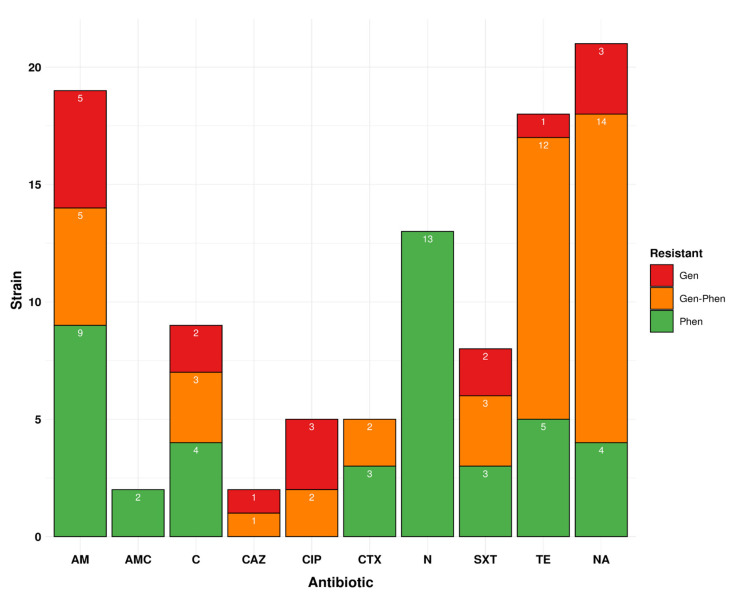
Individual antibiotic resistance profile of Peruvian isolates and their genotype–phenotype correlations for our ten tested antibiotics. Antibiotic abbreviation: ampicillin (AM), chloramphenicol (C), ciprofloxacin (CIP), trimethoprim-sulfamethoxazole (SXT), cefotaxime (CTX), nalidixic acid (NA), amoxicillin-clavulanate (AMC), nitrofurantoin (N), tetracycline (TE), and ceftazidime (CAZ). AMR profile abbreviation: Gen, genotypic; Phen, phenotypic; Gen-Phen, genotypic-phenotypic.

Out of the 90 Peruvian samples, 44 isolates (48.9%) contained resistance to at least one drug from the tested drugs using the disc diffusion assay. Out of these 44 isolates, the highest phenotypic resistance profile was for NA (*n* = 18, 40.9%; or 20.0% considering total 90 isolates), TE (*n* = 17, 38.6% or 18.9% considering total 90 isolates), AM (*n* = 14, 31.8%; or 15.6% considering total 90 isolates), N (*n* = 13, 29.6%; or 14.4% considering total 90 isolates), and 11 samples (25.0%; or 12.0% considering total 90 isolates) show MDR (Supplementary Table S1, Figure 4). These MDR isolates mostly belong to SC8 (*n* = 5), followed by SC9 (*n* = 2), SC15 (*n* = 2), SC17 (*n* = 1). In sample collection site-based analysis, Lima shows most (*n* = 10) of the MDR isolates and only one from Huánuco (Supplementary Table S1).

### 3.5. Genotypic Profile Showed 31.0% of Drug Resistance Isolates Are Circulating in Peru

The genotypic profile for 90 isolates showed the presence of a total of 63 (chromosomal + plasmid) different AMR genes as per drug class and resistance mechanisms. We found 31 were chromosomal, 32 were acquired in the 90 samples (Supplementary Table S3). However, since we have only the DNA sequence data, we over-ruled the expression-based drug resistance mechanism and have considered only the genes that confer drug resistance if it is present or when alleles were present. Based on these criteria, we found 19 isolates containing mobile AMR genes, two isolates harboured known single nucleotide variations in chromosomal AMR genes that conferred drug resistance, and seven isolates contained both the mobile AMR gene as well as variations in chromosomal AMR genes (Supplementary Table S3). Therefore, 28 isolates (31.0%) showed resistance to at least one drug gene/variation, including an untested drug.

While we considered the use of 10 antibiotics and DNA-based criteria, we found 26 isolates that have at least one AMR gene/variation in the genome. Out of these 26 isolates, 17 isolates contained mobile AMR genes, two isolates had known variations in chromosomal AMR genes, and seven isolates had both the mobile AMR gene and variations in chromosomal AMR genes (Supplementary Table S3). In this analysis, the resistance genotypic profile was observed: nalidixic acid was (*n* = 17, 65.4% or 18.9% considering total 90 isolates), tetracycline (*n* = 13, 50.0% or 14.4% considering total 90 isolates), and ampicillin (*n* = 10, 11.0%) (Figure 4). These MDR isolates mostly belong to SC8 (*n* = 5) and SC9 (*n* = 3) (Supplementary Table S3). The details of the mobile AMR gene and variations in chromosomal AMR genes of our samples are given in Table 1 and Table S3.

### 3.6. Ciprofloxacin and Ceftazidime Resistance Shows the Best Genotype–Phenotype Correlation, but Nitrofurantoin Does Not

While we compared our genotype-based drug resistance isolates to the phenotype data for ten tested antibiotics, we observed that 22 isolates were phenotypically resistant; they did not contain a known AMR plasmid or mobile gene or any mutation in the chromosomal AMR gene. On the other hand, out of the 28 genotypically resistant isolates, four isolates did not demonstrate phenotypically correlation to NA (*n* = 2), multi-drug resistance (*n* = 2) and additional drugs not testing (*n* = 2). Therefore, we considered a total of 50 isolates (44 phenotypically and 4 genotypically resistant to 10 tested drugs, and 2 genotypically resistant to untested antibiotics) for our further analysis (Table 2).

While we considered the genotype and phenotype data for ten drugs, 22 (*n* = 44–22) isolates had no correlation between the genotype and phenotype. In these 22 phenotypically drug-resistant isolates, ten isolates showed resistance exclusively to N, 3 to AM, 2 to CTX, and one each to NA, TE, AMC, AM + N, AM+ CTX+ C, AM + C+ TE, and AM+ SXT (Table 2, Supplementary Table S3). According to our calculation, as described in the method, the genotype–phenotype correlations for the ten tested antibiotics, the accuracy was 84.4% to 98.9%, specificity is between 93.4% and 100.0%, and the sensitivity reached up to 100.0% only for ciprofloxacin and ceftazidime resistance (Table 1). Ciprofloxacin and ceftazidime show >96.0% accuracy, specificity, sensitivity, and the lowest values were for beta-lactam resistance, followed by chloramphenicol and trimethoprim-sulfamethoxazole. In addition to that, we did not obtain a good sensitivity for nitrofurantoin as most of the phenotypical nitrofurantoin resistance isolates do not have any known nitrofurantoin resistance marker (Table 1).

### 3.7. Seventeen Different Plasmids Carrying 30 AMR Genes Were Identified in Peruvian Isolates

**Figure 5:**
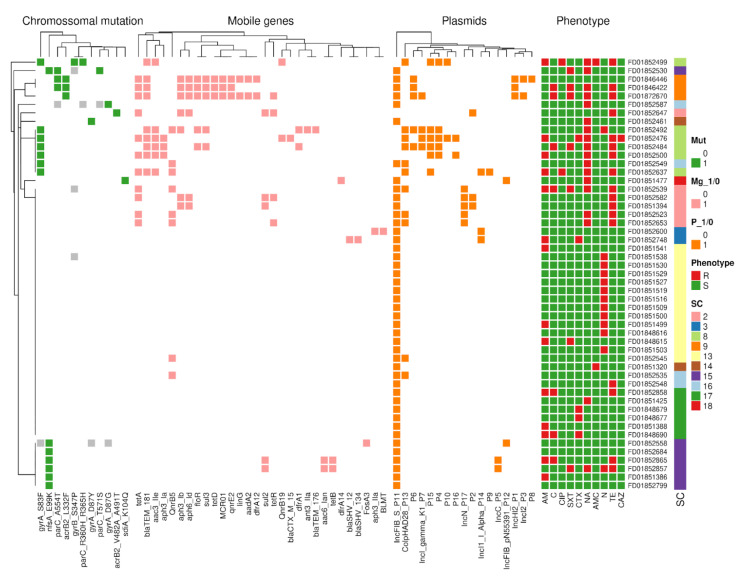
Heatmap showing presence and absence of antimicrobial resistance (due to chromosomal AMR gene variations, mobile genes, and plasmids), their corresponding phenotype, and sequence cluster classification in 50 *Salmonella* Typhimurium isolates.

We identified a total of 47 different plasmids in the 90 Peruvian isolates of which 30 plasmids did not carry any AMR gene, while 17 contained at least one AMR gene. A total of 30 AMR genes were found in the 17 plasmids from 28 isolates. A maximum occurrence of eight AMR genes was found in one plasmid (lncHI2A family, isolate-FD01846422) (Supplementary Table S4). These isolates mainly belong to SC9, SC2 and SC8 lineages. Among the 90 isolates, 83 isolates had the lncFIB virulence plasmid, and this lncFIB plasmid was observed in all the sequencing clusters except the isolates that belong to SC18 and one isolate under SC2 (Supplementary Table S1, Figure 5). The details of the isolates and their corresponding plasmid AMR genes are given in Table 3 and Table S4.

In addition, we detected plasmid-mediated resistance to third-generation cephalosporin, lncI-gamma/k1_P7 plasmid (*n* = 1/SC8) carrying *blaCTX-M-15* gene and IncI1-I(Alpha)_P14 plasmid (*n* = 1/SC3), *blaSHV-12* and *blaSHV-134* genes (Supplementary Table S4). Eleven isolates (*n* = 5/SC8, *n* = 3/SC2, *n* = 2/SC16, and *n* = 1/SC13) showed presence of Col(pHAD28) plasmid carrying fluoroquinolone-resistant either *qnrB19* or *qnrB5* gene. The fluoroquinolone-resistant *qnrE2* gene was found in three isolates belonging to the SC9 group that contained the IncHI2_P1 plasmid (Supplementary Table S4). Another critically important antibiotic-resistant gene, *fosA3*, for fosfomycin was identified in IncFIB(pN55391)_P12 (*n* = 1/SC15), and *mcr-1* gene for colistin resistance was found in IncHI2_P1 (*n* = 1/SC9), lncI2_P3 (*n* = 2/SC9) (Supplementary Table S4).

**Table 2:** Profile of 50 Peruvian drug resistance isolates (44 phenotypically and 4 genotypically resistant to 10 tested drugs, and 2 genotypically resistant to untested antibiotics).

SC	Isolate	Resistance Genes Profile	Resistance Plasmids Profile	Resistance Phenotypic Profile	Institution/Hospital	City	Year
**SC9**	FD01846446	*tetA*, *tetD*, *sul3*, *linG*, *floR*, *dfrA12*, *blaTEM-181*, *qnrE2*, *mcr-1*, *aph(6)-Id*, *aph(3″)-Ib*, *aadA2*, *dfrA1*	IncHI2_P1, IncI2_P3, P6, P8, IncFIB(S)_P11	-	Hospital Emergencias Pediátricas	Lima	2017
	FD01846422	*tetA*, *tetD*, *sul3*, *linG*, *floR*, *dfrA12*, *blaTEM-181*, *qnrE2*, *mcr-1*, *aph(6)-Id*, *aph(3″)-Ib*, *aadA2*, *dfrA1*, *sul2*	IncHI2_P1, P6	NA, C, SXT, TE	Hospital Emergencias Pediátricas	Lima	2017
	FD01872670	*tetA*, *tetD*, *sul3*, *linG*, *floR*, *dfrA12*, *blaTEM-181*, *qnrE2*, *mcr-1*, *aph(6)-Id*, *aph(3″)-Ib*, *aadA2*, *dfrA1*, *sul2*, *tetR*	IncHI2_P1, IncI2_P3, P6, P8, IncFIB(S)_P11	NA, C, SXT, TE	Hospital Emergencias Pediátricas	Lima	2017
**SC8**	FD01852492	*aac(3)-Iie*, *blaTEM-181*, *gyrA* p.S83Y, *aph(3″)-Ib*, *dfrA1*, *floR*, *qacL*, *sul3*, *qnrB5*, *ANT(3″)-Iia*, *blaTEM-176*	P4, IncI-gamma/K1_P7, P6, Col(pHAD28)_P13	NA, N	LRR Apurimac	Apurimac	2015
	FD01852476	*aac(3)-Iie*, *blaTEM-181*, *gyrA* p.S83Y, *aph(3′)-Ia*, *tetA*, *qnrB19*, *blaCTX-M-15*	P4, IncI-gamma/K1_P7, P10, Col(pHAD28)_P13, P16	NA, TE, AM, CTX, CAZ	INSN	Lima	2015
	FD01852484	*aac(3)-Iie*, *blaTEM-181*, *gyrA* p.S83Y, *aph(3′)-Ia*, *dfrA1*, *floR*, *qacL*, *sul3*	P4, P6, IncI-gamma/K1_P7, Col(pHAD28)_P13	NA, C, SXT	Hospital Emergencias Pediátricas	Lima	2015
	FD01852637	*aac(3)-Iie*, *blaTEM-181*, *gyrA* p.S83Y, *tetA*, *qnrB5*	P9, Col(pHAD28)_P13	NA, CIP, TE, AM	Hospital Emergencias Pediátricas	Lima	2015
	FD01852500	*aac(3)-Iie*, *blaTEM-181*, *gyrA* p.S83Y, *aph(3′)-Ia*, *tetA*	P4, P15, P16	NA, TE, AM	INSN	Lima	2016
	FD01852499	*aac(3)-Iie*, *blaTEM-181*, *gyrA* p.S83Y, *qnrB19*	P4, Col(pHAD28)_P13	NA, CIP, AM, AMC	Hospital Emergencias Pediátricas	Lima	2016
**SC2**	FD01852647	*aph(3″)-Ib*, *aph(6)-Id*, *sul2*, *tetA*, *tetR*	P2	TE	INEN	Lima	2015
	FD01852582	*aph(3″)-Ib*, *aph(6)-Id*, *sul2*, *tetA*, *tetR*	P2, IncN_P17	TE	Hospital Emergencias Pediátricas	Lima	2013
	FD01851394	*aph(3″)-Ib*, *aph(6)-Id*, *sul2*, *tetA*	P2	TE	Hospital Emergencias Pediátricas	Lima	2013
	FD01852653	*qnrB5*, *tetA*, *tetR*	Col(pHAD28)_P13, IncN_P17	NA, TE	DISA Lima Ciudad	Lima	2012
	FD01852523	*qnrB5*, *tetA*	IncFIB(S)_P11, Col(pHAD28)_P13	NA, TE	INEN	Lima	2016
	FD01852539	*qnrB5*, *tetA*	IncFIB(S)_P11, Col(pHAD28)_P13	NA, TE, AM, C, SXT	Hospital Emergencias Pediátricas	Lima	2016
**SC18**	FD01851477	*aph(3′)-Ia*, *dfrA14*	IncFIB(pN55391)_P12	NA	DIRESA Trujillo	La Libertad	2008
**SC16**	FD01852549	*qnrB5*, *gyrA* p.S83F	Col(pHAD28)_P13	NA	UNMSM	Lima	2017
**SC16**	FD01852587	*gyrA* p.D87Y	-	NA	INEN	Lima	2014
**SC14**	FD01852461	*gyrA* p.D87Y	-	NA	INEN	Lima	2015
**SC16**	FD01852535	*qnrB5*	Col(pHAD28)_P13	-	Cusco	Cusco	2016
**SC13**	FD01852545	*qnrB5*	Col(pHAD28)_P13	-	INEN	Lima	2017
**SC15**	FD01852865	*sul2*, *tetB*, *aac(6′)-Ian*	IncC_P5	AM, C, TE	Hospital Dos de Mayo	Lima	2010
	FD01852857	*sul2*, *tetB*, *aac(6′)-Ian*	IncC_P5	STX, NA, TE, N	DIRESA Huanuco	Huanuco	2010
	FD01852558	*fosA3*	IncFIB(pN55391)_P12	*NOT TESTED*	DIRESA Callao	Callao	2013
	FD01852530	*KpnH*	-	SXT, NA	INEN	Lima	2016
**SC13**	FD01851538	*KpnH*	-	N	INEN	Lima	2016
**SC3**	FD01852748	*blaSHV-12*, *blaSHV-134*	IncI1-I(Alpha)_P14	AM, CTX	INEN	Lima	2011
	FD01852600	*aph(3′)-Iia*, *BLMT*	IncI1-I(Alpha)_P14	*NOT TESTED*	INEN	Lima	2014
**SC17**	FD01851425	-	-	NA	CENAN/INS	Lima	2007
**SC16**	FD01852548	-	-	TE	Hospital Emergencias Pediátricas	Lima	2017
**SC13**	FD01851503	-	-	N	DIRESA Trujillo	La Libertad	2005
	FD01848616	-	-	N	LIMA CIUDAD	Lima	2005
	FD01851500	-	-	N	Puno	Puno	2005
	FD01851509	-	-	N	Direccion de Salud I	Callao	2005
	FD01851516	-	-	N	LRR Chiclayo	Lambayeque	2005
	FD01851519	-	-	N	Huaraz	Huaraz	2005
	FD01851527	-	-	N	Direccion de Salud I	Callao	2006
	FD01851529	-	-	N	Huaraz	Huaraz	2006
	FD01851530	-	-	N	INEN	Lima	2006
	FD01851538	-	-	N	LIMA	Lima	2006
**SC17**	FD01848677	-	-	CTX	Cajamarca	Cajamarca	2000
	FD01848679	-	-	CTX	LIMA ESTE	Lima	2000
	FD01851388	-	-	AM	INEN	Lima	2010
**SC15**	FD01851386	-	-	AM	Hospital Emergencias Pediátricas	Lima	2010
**SC13**	FD01851541	-	-	AM	Hospital Santa Maria Del Socorro	Ica	2006
**SC14**	FD01851320	-	-	AMC	CENAN/INS	Lima	2009
**SC13**	FD01851499	-	-	AM, N	LRR Chiclayo	Lambayeque	2005
**SC13**	FD01848615	-	-	AM, SXT	Hospital San Bartolomé	Lima	2005
**SC17**	FD01852858	-	-	AM, C, TE	DISA Lima Ciudad	Lima	2010
**SC17**	FD01848690	-	-	AM, C, CTX	Hospital San Bartolomé	Lima	2001

Antibiotic abbreviation: ampicillin (AM), chloramphenicol (C), ciprofloxacin (CIP), trimethoprim-sulfamethoxazole (SXT), cefotaxime (CTX), nalidixic acid (NA), amoxicillin-clavulanate (AMC), nitrofurantoin (N), tetracycline (TE), and ceftazidime (CAZ).

### 3.8. Hospital Emergencias Pediatrica Shows the Presence of Most of the MDR Isolates in Peru

Out of the 26 genotypically AMR isolates, 21 were from Lima, and 1 was from Apurimac, La libertad, Callao, Huanuco, and Cusco. Of these twenty-one AMR isolates from Lima, ten are MDR isolates, with seven found at Hospital Emergencias Pediatrica, two found at Instituto Nacional de Salud del niño, and one is present in LRR Apurimac. Out of the phenotypically resistant 44 isolates, 12 were MDR (Lima *n* = 11, Huanuco *n* = 1). From these twelve MDR isolates, six isolates were from Lima, Hospital Emergencias Pediatrica, two were from Instituto Nacional de Salud del niño and another four MDR isolates were distributed in four different hospitals in Lima. AM, NA and TE resistance were the most prevalent in these MDR isolates. Three isolates showed phenotypic resistance to maximum five antibiotics (FD01852539: AM + C + SXT + NA + TE; FD01852476: AM + CTX + NA + TA + CAZ; and FD01852499: AM + CIP + NA + AMC + TE) and were prevalent at Hospital Emergencias Pediatrica and Instituto Nacional de Salud del niño. Importantly, we also found that *Salmonella* acquired MDR genes after 2015 (Table 2 and Table S3).

### 3.9. Probable NA Resistance New Single Nucleotide Variations from Core AMR Gene Analysis

Based on the core genome analysis, we identified 30 non-reported non-synonymous single nucleotide variations (SNVs) in 11 AMR genes in our samples. Among these, four aminoglycoside resistance genes (*acrB1*, *kdpE*, *mdtB*, *mdtC*) had 17 SNVs and the microcin resistance gene (*yojI*) showed two SNVs. However, we were unable to proceed with these variations as we did not perform the phenotypic tests for these drugs (Supplementary Tables S3 and S5). The other 11 new SNVs detected in 6 AMR genes (emrB, parC, gyrB, acrB2, sdiA, nfsA) are associated with fluoroquinolone, multiclass, and nitrofurantoin resistance were selected for further analysis. The isolates that showed a specific drug resistance phenotype but had no known marker for that phenotype but had a new variation were selected. Following this strategy, finally, a total of three SNVs in two genes *parC* (A554T, T571S for NA), and *sdiA* (K104Q for NA), were selected for structure-based functional annotation (Supplementary Table S5).

### 3.10. Probable Nitrofurantoin Resistance Eight New Genes and Their Variations from GWAS Analysis

Out of the 44 phenotypically resistant isolates, 13 isolates showed N resistance but had no known AMR markers for N. Therefore, in the GWAS analysis, we focused on these 13 isolates to predict the potential causal SNVs associated with N resistance. We observed eight non-synonymous substitution variations in eight different genes in eleven isolates that were significantly associated with N resistance (p < 0.0000526). It is also important to note that these 11 isolates belong to SC13, and the SC13 group had 24 isolates. Therefore, any SC13 isolate containing all eight variations has a 46.0% chance of showing the N-resistant phenotype. The SNVs were MdtH L15P, hypothetical protein G6V, QseC1 L19V, PpnN R116C, BioH A236T, WecA B284I, PurA A103G, and Tsr2 D161G. The details of these genes and SNVs are provided in Table 3. The first logistic regression-based negative results (false positive) of N resistance association are shown in Supplement Table S6.

**Table 3:** Potential nitrofurantoin resistance eight genes and their SNVs identified with GWAS.

Locus	Gene	Product	Effect	*p*-Value
NC_003197.2:1252216	*mdtH*	Multi-drug resistance protein MdtH	missense_variant c.44T > C p. Leu15Pro	1.85112239616509e-06
NC_003197.2:2585776		hypothetical protein	missense_variant c.17G > T p. Gly6Val	3.9922233235481e-06
NC_003197.2:2933724	*qseC_1*	Sensor protein QseC	missense_variant c.55A > C p.Ile19Leu	1.85112239616509e-06
NC_003197.2:3119194	*ppnN*	Pyrimidine/purine nucleotide 5′-monophosphate nucleosidase	missense_variant c.346C > T p.Arg116Cys	1.85112239616509e-06
NC_003197.2:3667964	*bioH*	Pimeloyl-[acyl-carrier protein] methyl ester esterase	missense_variant c.706G > A p.Ala236Thr	1.85112239616509e-06
NC_003197.2:4127935	*wecA*	Undecaprenyl-phosphate alpha-N-acetylglucosaminyl 1-phosphate transferase	missense_variant c.850G > A p.Val284Ile	1.85112239616509e-06
NC_003197.2:4609418	*purA*	Adenylosuccinate synthetase	missense_variant c.308C > G p.Ala103Gly	1.85112239616509e-06
NC_003197.2:4790581	*tsr_2*	Methyl-accepting chemotaxis protein I	missense_variant c.482A > G p.Asp161Gly	1.85112239616509e-06

### 3.11. Functional Annotation of Nalidixic Acid and Nitrofurantoin Resistance Possible New Variations

We tested the effect of the newly identified three SNVs from two core AMR genes (for NA resistance) and eight new SNVs from eight genes (N resistance) from our GWAS analysis following the methods we described.

We determined the 3D structure of all these proteins, except the hypothetical protein and *tsr_2*. Therefore, we succeed in checking the effect of twelve new SNVs in nine genes (Supplementary material S1). Out of the four known fluoroquinolone resistance SNVs in *gyrA*, we found three SNVs that are destabilising (Table 4), indicating that many of the variations that have destabilising effects may be associated with drug resistance. Only the new SNVs for *parC* p.T571S, A554T, and *sdiA* p.K104Q were present in strains phenotypically resistant to NA and N, respectively, with no other known resistance gene. While we tested the effect of the identified unknown SNVs in other genes, except *ppnN* p.R116C, all 11 new SNVs were found to destabilise their corresponding protein. Therefore, considering the destabilising effect of the *gyrA* mutations, we may conclude that our identified new SNVs may be associated with NA and N resistance (Table 4).

## 4. Discussion

Our study is the first using WGS analysis and antimicrobial susceptibility test (AST) of *S.* Typhimurium isolates (*n* = 90) between 2000 and 2017 from different cities in Peru to examine the diversity, WGS-AST correspondence, resistome profile, and emerging lineages. Our study concludes that a considerable nucleotide, gene, and phylogenetic group diversity circulates in Peru.

Peruvian samples belong predominant to ST19, with a diverse accessory content, constituted by plasmids and segregated by phylogenetic groups. We reported that 94.4% of *S.* Typhimurium isolates in Peru belong to sequence type ST19. This was expected because ST19 is among the most predominant ST associated with gastroenteritis cases worldwide, and it is the most ancestral and diverse phylogenetically ST for the serovar Typhimurium [11,19,20].

The population contained an open pan-genome with many repertoire genes for global and Peruvian isolates comparable to previous reports [19,63,64]. However, the core gene content was smaller than previously reported (3672 genes) [19] Fu et al. (3846 genes) [64]. Likewise, we defined the accessory genome as constituted of diverse plasmids without predicting other mobile elements. Other studies highlight that the composition of the accessory genome for *S.* Typhimurium is mainly based on the diversity contribution of prophage genes, up 23.4%, followed by other elements such as plasmids and mobile islands, up 13.3% [19,64]. In addition, we did not corroborate that phylogenetic groups segregate the accessory genome by PCA analysis (analysis not shown).

Initially, a low accumulation nucleotide of 400–600 SNPs was reported for the serovar [20]. Although, other studies showed a large accumulation of SNPs by isolate (1232 SNPs) [64] and a total of 62,884 SNPs for the African population [10]. These studies are comparable to the vast number of polymorphisms (3045 SNPs) within genomes circulating in Peru with a moderate median pi value (0.135) at the intrapopulation diversity level described by Pons, 1996 [65].

The phylogenetic topology showed two high-order clades and population structures identified at least 10 lineages supported by subclades with depth branches to multiple branches. This topology has already been discussed previously in studies; this includes an alpha clade basal with livestock samples with a diversity of terminal branches corresponding to clonal expansions. A distinct beta clade is characterised by multiple lineages from the vast host (including wild avian) that are deeply rooted [19,20]. Likewise, we report the emergence of SC13 and SC17 lineages by their relative widespread in the country and the prevalent MDR phenotype of SC9 and SC8 lineages. It has already been shown that subclades are under different anthropogenic selection pressures. Antibiotic use might provide the selection pressure driving the emergence of sub-lineages containing AMR genes [10,11,19,66]. MDR strains harbour variable numbers of resistant plasmids reported mainly from livestock samples and outbreaks in hospitals and foods [19,20].

*S.* Typhimurium presents considerable resistance to first-line antibiotics, susceptibility to cephalosporins and ciprofloxacin, and low sensitivity value of phenotypic prediction due to the unknown resistance mechanism. This study explored resistance trends, transmission, profile genotypic and phenotypic, correlation, and discovery of new genetic bases for resistance phenotypes with phylogeny. The study reports a high number of strains (48.8%) with phenotypic resistance to at least one antibiotic compared to the variable prevalence (26.0%/n = 95, 61.6%/n = 193, 37.3%/n = 3491) for NTS clinical and food samples from Peru, US, and England, respectively [21,22,67]. We found considerable resistance to first-line antibiotics (nitrofurantoin, tetracycline, nalidixic acid, and ampicillin); only 12.0% were MDR strains. The persistence of resistance to first-line antibiotics over the years in this study was expected due to the continued use of treatment in *Salmonella* [24].

Previous studies of NTS and *S.* Typhimurium strains show a significant number (24.3%-43.0%) of MDR strains to first-line antibiotics except for nitrofurantoin, and with the addition of sulphonamide, streptomycin, and chloramphenicol (in some cases) in American Latin, USA, and England [21,22,24,66,68]. This minor prevalence of MDR strains compared to other studies of NTS samples should include different serovars that present high and diverse MDR. In recent years, *S.* Infantis has been the most predominant serovar detected in clinical samples associated with high resistance to first-line antibiotics, third-generation cephalosporins [7,8], and ciprofloxacin [8,68,69]. Although 85.0% of our samples were clinical isolates, *S.* Typhimurium, compared to other serovars, still presents a reduced resistance to priority antibiotics. On the contrary, *S.* Typhimurium is a relevant pathogen in guinea pigs with distinct and moderate AMR profiles that include colistin and enrofloxacin [70], erythromycin and nitrofurantoin resistance [71]. In addition, *Salmonella* isolates from chicken meat show high quinolone resistance (enrofloxacin and NA). Currently, the resistance spectra of the MDR strains of *Salmonella* serovars have been emerging in farm animals [24]. Because quinolones, chloramphenicol, aminoglycosides, and nitrofurantoin are exhaustively used as treatment and/or prophylactic in farm animals [68,70,71,72,73,74], constituting the leading resource of transmission on the emergence of resistant strains is reported.

WGS showed the content of AMR chromosomal genes with an impact on the resistance based on their expression. These AMR genes encoded most efflux pumps reported by Seribelli [11]. Complementary, we identified 32 AMR acquired genes (Table 1, this profile AMR genes were similar and minor to previous works [21,22,23,66,73]. These studies detecting additionally other EBSLs, ribosomal protection mechanisms, PMQR genes, phosphotransferase, efflux pump, and an acetyltransferase that confer resistance to spectrum extended beta-lactam, tetracycline, fluoroquinolone, macrolides, and phenicol, respectively. Genotypic AMR profile to priority antibiotics shows the nalidixic acid resistance associated with the presence of PMQR genes (*qnrB5*, *qnrE2*, *qnrB19*) or mutations (D87G, D87Y, S83F, S83Y) in the *gyrA* gene, and presence combined of both markers are associated with ciprofloxacin resistance [21]. Interestingly, the considerably reduced ciprofloxacin susceptibility did not exhibit these markers. This is because the efflux pump’s overexpression was related to intermediate resistance [75], and the disc diffusion test did not adequately detect reduced susceptibility to fluoroquinolones [76]. In only two strains, extended-spectrum beta-lactam resistance was associated with three EBSLs genes (*blaCTX-M-15*, *blaSHV-12*, *blaSHV-134*). Additional WGS allowed the detection of genes associated with additional drug resistance as aminoglycoside, aminocoumarin, bacitracin, nitroimidazole, microcin, lincosamide, bleomycin, fosfomycin, colistin, and multiclass.

A worrying trend is an increase in resistance to treatment antibiotics (extended-spectrum cephalosporins and ciprofloxacin) for *Salmonella*. Despite them, our study shows susceptibility to ciprofloxacin and third-generation cephalosporins, reported in other works [21,22,24,66,67,68,73] without co-resistance to both antimicrobial classes. Whereby the use of third-generation cephalosporins for treatment in *S.* Typhimurium infections would be recommended.

WGS strategy allows monitoring and complementing the prediction of phenotypic AMR profiles [23,73]. We found good accuracy values (prediction of true positives and negatives) and specificity (the absence of known markers predicting true negatives). Comparable successful correlation values were reported, 99.0% [73], 97.8% [21], 95.4% [22], 89.9% [23], and 85.4% [69]. Nonetheless, we found the best sensitivity values to predict resistance to only ceftazidime and ciprofloxacin and the highest discrepancy values to predict resistance to beta-lactam, chloramphenicol, SXT, and nitrofurantoin. Previous work also found discord in the prediction of resistance to beta-lactams [66,73], sulfamethoxazole [23], ciprofloxacin [73] and tetracycline [66]. Due to the high number of contradictions between genotype–phenotype compared with previous reports cannot conclude that this would be an alternative method of predicting antimicrobial susceptibility.

Mismatch categories with a lower sensitivity, where an isolate is genotypically predicted to be susceptible but exhibits phenotypic resistance, highlights limitations based on sequence quality [77], partial assemblies, lack of updated AMR database, and rely on the prediction based only on the genome [12,78,79]. Continuous findings should be carried out to identify novel resistance mechanisms and be incorporated into the reference databases to maintain a high level of prediction sensitivity. Other inconsistent results support the need for combined AST strategies, such as the microdilution test, an efficient method with quantitative results [28]. This study also highlights the performance of the routine antibiotic susceptibility test and works with a balanced number of phenotypic samples and population representations [80].

The diversity of plasmid families carrying AMR genes in isolates belong to SC8 and SC9 lineages and was detected in two hospital centres considered the focus of transmission of antimicrobial resistance. WGS also allows identifying AMR genes commonly present on plasmids, primary transmission resources, and related to the emergency lineages. Here, we reported at least 17 resistant plasmid families, including conjugative and mobilisable plasmids, with the potential threat of spreading AMR genes in NTS [14,22]. These resistance plasmids belong to F, ColE, I1, C, HI1, HI2, and N families, such as a previous work of *Salmonella* isolates from food animals in the USA predicted 212 resistance plasmids [22]. In Peru, a conservative virulence plasmid (pSV) in *S*. Typhimurium [67] and MDR Mega plasmid in *S.* Infantis [8] have been described. In Peru, *S.* Typhimurium isolates are prevalent that contains the virulence plasmid (pSV) [67,81]. However, the absence of plasmids in the phylogenetic group could be due to the competence with other AMR plasmids or other genetic and environmental factors that modulate the residence [81].

Interestingly, the genotypic profile is linked to family plasmid in some lineages. For example, two only strains contain replicon plasmid carrying ESBLs genes and unnamed replicon plasmid P4 carrying beta-lactamases genes in lineage SC8. Lineages S8 and SC2 harbour PMQR genes in Col(pHAD28) plasmid. Col(pHAD28) plasmids related to fluoroquinolone resistance were reported [82]. *MCR-1*-carrying lncI2 and IncHI2 belong to the SC9 lineage. These dominant mobilisable plasmids show colistin resistance [83,84]. Likewise, only a strain carries *fosA3*-carrying IncFIB plasmid. Previous studies have reported antibiotic’s last line resistance as the *fosA3* gene in AMR plasmids [85] and IncFIB plasmids [86,87]. Intriguingly, SC9 isolates harbour the IncHI2 plasmid that carries many AMR genes. This plasmid is a dominant mobilisable detected among MDR *Salmonella*, playing a role in the acquisition of ARGs, and has been reported recently encoding ESBLs [10,88,89,90]. Thus, active surveillance is needed to minimise the global spread of lncIA-I(Alpha), IncI-gamma/K1, lncFIB, lncI2, and IncHI2 resistant plasmids link an SC9, SC8, and SC2 lineage. MDR strains were found mainly from the Hospital de Emergencias Pediátricas, and Instituto Nacional de Salud del Niño. The previous report shows the presence of MDR isolates from serovar Infantis in the Hospital de Emergencias Pediátricas [91]. Therefore, antimicrobial screening routines should be implemented to mitigate the spread of these healthcare-associated MDR strains in Peruvian hospitals.

GWAS analysis allows us to identify new non-synonymous mutations that can potentially improve resistance fitness; however other resistance confirmation strategies are necessary. We reported new SNVs in *parC* p.R360H, R365H gene, and *sdiA* p.K104Q that show destabilising effect protein with a possible impact on the protein function. Mutations in *gyrA-parC* genes decrease the binding affinity of quinolones with DNA-topoisomerase enzymes [92,93]. Efflux pumps are encoded in chromosomes and play intrinsic roles in multi-drug resistant Gram-negative bacteria [25]. *sdiA* gene acts as a positive regulator of the constitutive expression of the AcrAB–TolC pump system [94]. Nucleotide variations in this regulator [25] act in high-level fluoroquinolone resistance and other antimicrobials [94,95]. Genetic variation targets could result in overexpression of these proteins. For instance, SNVs in *acrR* regulator or multi-drug pump AcrAB were associated with high-level fluoroquinolone resistance [94].

We found that considerable nitrofurantoin resistance could not be associated with known AMR markers such as those previously reported [96]. This would happen because the ARM databases have few AMR genes since the mechanisms of action of nitrofurans are poorly studied [94]. Thereby, GWAS analysis identified eight non-synonymous substitutions potentially associated with resistance to nitrofurantoin; six show the destabilising effect protein. Genetic variation is within multi-drug efflux pump [97]; regulator purine homeostasis and biosynthesis [98]; biotin ring assembly [99]; pathway LPS O-antigen biosynthesis [100], and chemotactic-signal transducers added. However, we did not find that the mutational effect on these functional mechanisms could confer nitrofurantoin resistance. Likewise, these variants were present in the lineage SC13. To avoid spurious associations, GWAS analysis using the population stratification covariate was performed subsequently without associating any mutation to nitrofurantoin resistance. Hence, we suggest that the maintenance of these combined mutations in phylogenetic group strains would be fixed randomly and consequently could confer a resistance advantage.

## 5. Conclusions

Our work based on the WGS analysis has allowed us to understand the dynamics and determinants of antimicrobial resistance distinguished by the population diversity for *S.* Typhimurium. However, due to low sensitivity values from genotype–phenotype resistance correlation, it is still necessary to evaluate the use of WGS to predict AMR susceptibility. We recommend the third-generation cephalosporin antibiotic as a potential treatment against infection by *S.* Typhimurium. We can reinforce that WGS constitutes a complement but not an alternative to traditional methods to infer antimicrobial susceptibility, as a powerful tool that allows genome-based epidemiological study, monitoring AMR genes, virulence, plasmid typing, outbreaks, understanding of resistance mechanism, and transmission patterns. Pathogen genomic surveillance should be expanded globally and continuously monitored for better treatment of Salmonellosis and control strategies against the disseminating AMR. Future work should be based on better discordant prediction due to the absence of AMR genes on resistance phenotypes, adding new resistance mechanisms and improving the database’s reliability, replicating assay, large-scale samples, and supplementary technical methods.

## Figures and Tables

**Table 1:** The genotype to phenotype concordance using accuracy, sensitivity, and specificity for Peruvian isolates for ten tested drugs.

Antibiotic	New Variations in Chromosomal Genes	Known Variations in Chromosomal Genes	Mobile Resistance Genes	Susceptible Phenotype		Resistant Phenotype		Accuracy	Sensitivity	Specificity
				Resistant Genotype	Susceptible Genotype	Resistant Genotype	Susceptible Genotype			
				FP	TN	TP	FN	(TP + TN)/TOTAL	TP/(TP + FN)	TN/(TN + FP)
**Tetracycline (T)**	-	*-*	*tetA* (*n* = 15), *tetB* (*n* = 2), *tetD* (*n* = 3), *tetR* (*n* = 4)	1	72	13	5	94.4%	72.2%	98.6%
**Ampicillin (AM)**	*ompA* (*n* = 90), *ampH* (*n* = 46), *golS* (*n* = 90), *mdsA* (*n* = 87), *mdsB* (*n* = 90), *mdsC* (*n* = 69)	*-*	*blaTEM-176* (*n* = 1), *blaTEM-181* (*n* = 9), *blaSHV-134* (*n* = 1), *blaTEM-181* (*n* = 1), *blaCTX-M-15* (*n* = 1)	5	71	5	9	84.4%	35.7%	93.4%
**Amoxicillin-clavulanate (AMC)**		*-*	*blaCTX-M-15* (*n* = 1), *blaSHV-12* (*n* = 1), *blaSHV-134* (*n* = 1)	2	86	0	2	95.6%	0.0%	97.7%
**Cefotaxime (CTX)**		*-*		0	85	2	3	96.7%	40.0%	100.0%
**Ceftazidime (CAZ)**		*-*		1	88	1	0	98.9%	100.0%	98.9%
**Chloramphenicol (C)**	*golS* (*n* = 90), *mdsA* (*n* = 87), *mdsB* (*n* = 90), *mdsC* (*n* = 69)	*-*	*florR* (*n* = 5)	2	81	3	4	93.3%	42.9%	97.6%
**Ciprofloxacin (CIP)**	*mdtK* (*n* = 90), *crp* (*n* = 90), *emrA* (*n* = 90), *emrB* (*n* = 87) [p.G509D, G510D (*n* = 1)], *emrR* (*n* = 90), *gyrB* (*n* = 89) p.S347P (*n* = 1), *parC* (*n* = 89) [p.A554T (*n* = 3), p.R360H, R365H (*n* = 1), p.T571S (*n* = 1)]	*gyrA* (*n* = 90) p.S83Y (*n* = 6), p.S83F (*n* = 1), p.D87G (*n* = 1), p.D87Y (*n* = 1)	*qnrB5* (*n* = 8), *qnrE2* (*n* = 3), *qnrB19* (*n* = 2)	3	85	2	0	96.7%	100.0%	96.6%
**Nalidixic acid (NA)**				3	69	14	4	92.2%	77.8%	95.8%
**Trimethoprim-sulfamethoxazole (STX)**	-	*-*	*dfr1* (*n* = 5), *dfr12* (*n* = 2), *dfr14* (*n* = 1), *sul2* (*n* = 4), *sul3* (*n* = 5)	2	84	3	3	96.7%	50.0%	97.7%
**Nitrofurantoin (N)**	*nfsA* (*n* = 90), *nfsB* (*n* = 88), *ribE* (*n* = 89)	*-*	*-*	0	77	0	13	85.6%	0.0%	100.0%
**Aminoglycoside**	*aac(6′)-Iaa* (*n* = 90), *kdpE* (*n* = 74) [p.A115G, A115E (*n* = 2), p.L39P (*n* = 3), p.R81H, S100R (*n* = 1)]	-	*aac(3) IIe* (*n* = 6), *aph(3′)-Ia* (*n* = 4), *aac(3)-Iie* (*n* = 6), *aac(6′)-Ian* (*n* = 2), *ant(3″)-Iia* (*n* = 1), *aph(3″)-Ib* (*n* = 7), *aph(6)-Id* (*n* = 6)							
**Aminocoumarin and Aminoglycoside**	*baeR* (*n* = 90), cpxA (*n* = 86)	-	-							
**aminocoumarin**	*mdtB* (*n* = 84) [p.T69A (*n* = 5), p.S157A (*n* = 5), p.N199H (*n* = 1), p.R512H (*n* = 3), p.Q315R (*n* = 1), p.R590H (*n* = 5)], *mdtC* (*n* = 90) [p.T81S (*n* = 1), p.N113K (*n* = 1), p.N133D (*n* = 1)]	-	-							
**Multiclass**	*acrB2* (*n* = 90) [p.L332F (*n* = 3), p.V482A, A491T (*n* = 1)], *sdiA* (*n* = 90), *tolC* (*n* = 89), *H-NS* (*n* = 90), *marA* (*n* = 90), *acrB1* (*n* = 90) [p.T599P (*n* = 27), p.R418H (*n* = 4), p.L845F (*n* = 1)], *mdtC*, *marA*	-	*kpnH* (*n* = 3)							
**Bacitracin**	*bacA* (*n* = 89)	-	-							
**Nitroimidazole**	*msbA* (*n* = 90)	-	-							
**Microcin**	*yojI* (*n* = 90) [p.H431Y (*n* = 6), p.A366D (*n* = 2)]	-	-							
**Lincosamide**	-	-	*linG* (*n* = 3)							
**Bleomycin**	-	-	*BLMT* (*n* = 1)							
**Fosfomycin**	-	-	*fosA3* (*n* = 1)							
**Colistin**	-	-	*mcr-1* (*n* = 3)							

**Table 4:** Most of the non-synonymous variations for the NA and N resistance are predicted to be destabilising based on ΔΔG (Kcal/mol) calculation.

Analysis	Gene	Non-Synonymous Variations	Isolates	Prediction Stability ΔΔG (Kcal/mol)	Stability	Antibiotic Resistance
Variants in core AMR genes	***parC***	A554T	3	−1.82	Destabilising	Fluoroquinolone (NA, CIP)
		R360H, R365H	1	−1.86	Destabilising	
		T571S, A554T	1	−1.01	Destabilising	
	***gyrA***	D87G	1	−0.84	Destabilising	
		D87Y	1	0.18	Stabilising	
		S83F	1	−0.85	Destabilising	
		S83Y	6	−0.91	Destabilising	
	***sdiA***	K104Q	1	−0.18	Destabilising	Multiclass
	***nfsA***	E99K	7	−0.61	Destabilising	Nitrofurantoin
New SNPs using GWAS	***mdtH***	L15P	24	−1.34	Destabilising	
	**hypothetical protein**	G6V	24	-	-	
	***qseC_1***	I19L	24	−0.09	Destabilising	
	***ppnN***	R116C	24	0.24	Stabilising	
	***bioH***	A236T	24	−1.04	Destabilising	
	***wecA***	V284I	24	−0.47	Destabilising	
	***purA***	A103G	24	−1.19	Destabilising	
	***tsr_2***	N161G	24	-	-	

## Data Availability

Genome sequence data are available at the National Center for Biotechnology Information (NCBI). Metadata for samples, including accession, are included in Table S1.

## Supplementary Materials

The following supporting information can be downloaded at: https://www.mdpi.com/article/10.3390/antibiotics11091170/s1, Table S1: Epidemiological data, antimicrobial resistance profile, sequencing, typification, and genomic structure analysis from 90 *Salmonella* Typhimurium strains; Table S2: Epidemiological data, ST, and BAPS analysis from 140 *Salmonella* Typhimurium strains; Table S3: Genotypic and phenotypic resistance profiles of the 90 *Salmonella* Typhimurium strains; Table S4: AMR genes and plasmids characterization using Mob_suite; Table S5: Overview of known and new chromosomal mutations and phenotypic correspondence; Table S6: Top 20 ranked p-values from SNPs associated with nitrofurantoin resistance using Firth logistic regression; Figure S1: Whole-genome Average Nucleotide Identity (ANI) and sequence type; Figure S2: Nucleotide similarity and sequence type; Figure S3: Phylogenetic tree was generated using a maximum likelihood method with 1000 replicates of bootstrap using GTR+GAMMA to estimate evolutionary distance between 140 global isolates; Supplementary material S1: Model quality validation: The 3D structures were generated using SWISS-MODEL and evaluated using Ramachandram plot.

## References

[1] Havelaar A.H., Kirk M.D., Torgerson P.R., Gibb H.J., Hald T., Lake R.J., Praet N., Bellinger D.C., de Silva N.R., Gargouri N. (2015). World Health Organization Global Estimates and Regional Comparisons of the Burden of Foodborne Disease in 2010. PLoS Med..

[2] Tacconelli E., Carrara E., Savoldi A., Harbarth S., Mendelson M., Monnet D.L., Pulcini C., Kahlmeter G., Kluytmans J., Carmeli Y. (2018). Discovery, Research, and Development of New Antibiotics: The WHO Priority List of Antibiotic-Resistant Bacteria and Tuberculosis. Lancet Infect. Dis..

[3] Ministry of Health/Ministerio de Salud (MINSA) (2019). Boletin Epidemiologico Del Peru. Bol. Epidemiol. Del Peru.

[4] Ramirez-Hernandez A., Galagarza O.A., Álvarez Rodriguez M.V., Pachari Vera E., Valdez Ortiz MD C., Deering A.J., Oliver H.F. (2020). Food Safety in Peru: A Review of Fresh Produce Production and Challenges in the Public Health System. Compr. Rev. Food Sci. Food Saf..

[5] Zamudio M.L., Meza A., Bailón H., Martinez-Urtaza J., Campos J. (2011). Experiencias En La Vigilancia Epidemiológica de Agentes Patógenos Transmitidos Por Alimentos a Través de Electroforesis En Campo Pulsado (PFGE) En El Perú. Rev. Peru. Med. Exp. Salud Publica.

[6] Garcia C., Hinostroza N., Astocondor L., Ochoa T., Jacobs J. (2019). Characterization of ESBL-Producing *Salmonella enterica* Serovar Infantis Infection in Humans, Lima, Peru. Am. J. Trop. Med. Hyg..

[7] Sifuentes W.Q., Hurtado C.V., Meza A.M., Zamudio M.L., Gavilan R.G. (2020). Patterns of Resistance to Antimicrobials in Serovars of *Salmonella enterica* in Peru, 2012-2015. Rev. Chil. Infectol..

[8] Quino W., Hurtado C.V., Escalante-Maldonado O., Flores-León D., Mestanza O., Vences-Rosales F., Zamudio M.L., Gavilán R.G. (2019). Multidrogorresistencia de *Salmonella* Infantis En Perú: Un Estudio Mediante Secuenciamiento de Nueva Generación. Rev. Peru. Med. Exp. Salud Publica.

[9] Lan R., Reeves P.R., Octavia S. (2009). Population Structure, Origins and Evolution of Major *Salmonella enterica* Clones. Infect. Genet. Evol..

[10] Van Puyvelde S., Pickard D., Vandelannoote K., Heinz E., Barbé B., de Block T., Clare S., Coomber E.L., Harcourt K., Sridhar S. (2019). An African *Salmonella* Typhimurium ST313 Sublineage with Extensive Drug-Resistance and Signatures of Host Adaptation. Nat. Commun..

[11] Seribelli A.A., Gonzales J.C., de Almeida F., Benevides L., Cazentini Medeiros M.I., dos Prazeres Rodrigues D., de C. Soares S., Allard M.W., Falcão J.P. (2020). Phylogenetic Analysis Revealed That *Salmonella* Typhimurium ST313 Isolated from Humans and Food in Brazil Presented a High Genomic Similarity. Braz. J. Microbiol..

[12] Li X.Z., Nikaido H. (2009). Efflux-Mediated Drug Resistance in Bacteria: An Update. Drugs.

[13] Glenn L.M., Lindsey R.L., Frank J.F., Meinersmann R.J., Englen M.D., Fedorka-Cray P.J., Frye J.G. (2011). Analysis of Antimicrobial Resistance Genes Detected in Multidrug-Resistant *Salmonella enterica* Serovar Typhimurium Isolated from Food Animals. Microb. Drug Resist..

[14] Bogomazova A.N., Gordeeva V.D., Krylova E.V., Soltynskaya I.V., Davydova E.E., Ivanova O.E., Komarov A.A. (2020). Mega-Plasmid Found Worldwide Confers Multiple Antimicrobial Resistance in *Salmonella* Infantis of Broiler Origin in Russia. Int. J. Food Microbiol..

[15] Ellington M.J., Ekelund O., Aarestrup F.M., Canton R., Doumith M., Giske C., Grundman H., Hasman H., Holden M.T.G., Hopkins K.L. (2017). The Role of Whole Genome Sequencing in Antimicrobial Susceptibility Testing of Bacteria: Report from the EUCAST Subcommittee. Clin. Microbiol. Infect..

[16] Balouiri M., Sadiki M., Ibnsouda S.K. (2016). Methods for in Vitro Evaluating Antimicrobial Activity: A Review. J. Pharm. Anal..

[17] World Health Organization (WHO) (2020). GLASS Whole-Genome Sequencing for Surveillance of Antimicrobial Resistance: Global Antimicrobial Resistance and Use Surveillance System (GLASS).

[18] Zhang S., Li S., Gu W., Den Bakker H., Boxrud D., Taylor A., Roe C., Driebe E., Engelthaler D.M., Allard M. (2019). Zoonotic Source Attribution of *Salmonella enterica* Serotype Typhimurium Using Genomic Surveillance Data, United States. Emerg. Infect. Dis..

[19] Id M.B., Alikhan N.-F., Tan Thilliez Id G., Kirkwood Id M., Wheelerid N.E., Petrovska L., Dallman T.J., Adriaenssensid E.M., Hallid N., Kingsleyid R.A. (2020). Evolution of *Salmonella enterica* Serotype Typhimurium Driven by Anthropogenic Selection and Niche Adaptation. PLoS Genet..

[20] Branchu P., Bawn M., Kingsley R.A. (2018). Genome Variation and Molecular Epidemiology of *Salmonella enterica* Serovar Typhimurium Pathovariants. Infect. Immun..

[21] Neuert S., Nair S., Day M.R., Doumith M., Ashton P.M., Mellor K.C., Jenkins C., Hopkins K.L., Woodford N., de Pinna E. (2018). Prediction of Phenotypic Antimicrobial Resistance Profiles from Whole Genome Sequences of Non-Typhoidal *Salmonella enterica*. Front. Microbiol..

[22] McMillan E.A., Gupta S.K., Williams L.E., Jové T., Hiott L.M., Woodley T.A., Barrett J.B., Jackson C.R., Wasilenko J.L., Simmons M. (2019). Antimicrobial Resistance Genes, Cassettes, and Plasmids Present in *Salmonella enterica* Associated with United States Food Animals. Front. Microbiol..

[23] Mensah N., Tang Y., Cawthraw S., Abuoun M., Fenner J., Thomson N.R., Mather A.E., Petrovska-Holmes L. (2019). Determining Antimicrobial Susceptibility in *Salmonella enterica* Serovar Typhimurium through Whole Genome Sequencing: A Comparison against Multiple Phenotypic Susceptibility Testing Methods. BMC Microbiol..

[24] Wang X., Biswas S., Paudyal N., Pan H., Li X., Fang W., Yue M. (2019). Antibiotic Resistance in *Salmonella* Typhimurium Isolates Recovered from the Food Chain through National Antimicrobial Resistance Monitoring System between 1996 and 2016. Front. Microbiol..

[25] Tavío M.M., Aquili V.D., Poveda J.B., Antunes N.T., Sánchez-Céspedes J., Vila J. (2010). Quorum-Sensing Regulator SdiA and MarA Overexpression Is Involved in in Vitro-Selected Multidrug Resistance of Escherichia Coli. J. Antimicrob. Chemother..

[26] Farhat M.R., Freschi L., Calderon R., Ioerger T., Snyder M., Meehan C.J., de Jong B., Rigouts L., Sloutsky A., Kaur D. (2019). GWAS for Quantitative Resistance Phenotypes in Mycobacterium Tuberculosis Reveals Resistance Genes and Regulatory Regions. Nat. Commun..

[27] Mortimer T.D., Zhang J.J., Ma K.C., Grad Y.H. (2022). Loci for Prediction of Penicillin and Tetracycline Susceptibility in Neisseria Gonorrhoeae: A Genome-Wide Association Study. Lancet Microbe.

[28] Jorgensen J.H., Ferraro M.J. (2009). Antimicrobial Susceptibility Testing: A Review of General Principles and Contemporary Practices. Clin. Infect. Dis..

[29] Perez-Sepulveda B.M., Heavens D., Pulford C.V., Predeus A.V., Low R., Webster H., Schudoma C., Rowe W., Lipscombe J., Watkins C. (2020). An Accessible, Efficient and Global Approach for the Large-Scale Sequencing of Bacterial Genomes. Genome Biol..

[30] Andrews S. (2010). FASTQC A Quality Control Tool for High Throughput Sequence Data. Babraham Inst..

[31] Wick R.R., Judd L.M., Gorrie C.L., Holt K.E. (2017). Unicycler: Resolving Bacterial Genome Assemblies from Short and Long Sequencing Reads. PLoS Comput. Biol..

[32] Gurevich A., Saveliev V., Vyahhi N., Tesler G. (2013). QUAST: Quality Assessment Tool for Genome Assemblies. Bioinformatics.

[33] Simão F.A., Waterhouse R.M., Ioannidis P., Kriventseva E.V., Zdobnov E.M. (2015). BUSCO: Assessing Genome Assembly and Annotation Completeness with Single-Copy Orthologs. Bioinformatics.

[34] Seemann T. (2014). Prokka: Rapid Prokaryotic Genome Annotation. Bioinformatics.

[35] Hu Z., Wei C., Li Z., Tettelin H., Medini D. (2020). Computational Strategies for Eukaryotic Pangenome Analyses. Pangenome: Diversity, Dynamics and Evolution of Genomes.

[36] van Aggelen H., Kolde R., Chamarthi H., Loving J., Fan Y., Fallon J.T., Huang W., Wang G., Fortunato-Habib M.M., Carmona J.J. (2019). A Core Genome Approach That Enables Prospective and Dynamic Monitoring of Infectious Outbreaks. Sci. Rep..

[37] Noune C., Hauxwell C. (2017). MetaGaAP: A Novel Pipeline to Estimate Community Composition and Abundance from Non-Model Sequence Data. Biology.

[38] Robertson J., Nash J.H.E. (2018). MOB-Suite: Software Tools for Clustering, Reconstruction and Typing of Plasmids from Draft Assemblies. Microb. Genom..

[39] Carattoli A., Zankari E., Garciá-Fernández A., Larsen M.V., Lund O., Villa L., Aarestrup F.M., Hasman H. (2014). In Silico Detection and Typing of Plasmids Using Plasmidfinder and Plasmid Multilocus Sequence Typing. Antimicrob. Agents Chemother..

[40] Zhang S., den Bakker H.C., Li S., Chen J., Dinsmore B.A., Lane C., Lauer A.C., Fields P.I., Deng X. (2019). SeqSero2: Rapid and Improved *Salmonella* Serotype Determination Using Whole-Genome Sequencing Data. Appl. Environ. Microbiol..

[41] Jolley K.A., Bray J.E., Maiden M.C.J. (2018). Open-Access Bacterial Population Genomics: BIGSdb Software, the PubMLST.Org Website and Their Applications. Wellcome Open Res..

[42] Jain C., Rodriguez-R. L.M., Phillippy A.M., Konstantinidis K.T., Aluru S. (2018). High Throughput ANI Analysis of 90K Prokaryotic Genomes Reveals Clear Species Boundaries. Nat. Commun..

[43] Page A.J., Cummins C.A., Hunt M., Wong V.K., Reuter S., Holden M.T.G., Fookes M., Falush D., Keane J.A., Parkhill J. (2015). Roary: Rapid Large-Scale Prokaryote Pan Genome Analysis. Bioinformatics.

[44] Snipen M.L., Liland K.H. (2015). Micropan: An R-package for microbial pan-genomics. BMC Bioinform..

[45] Tettelin H., Masignani V., Cieslewicz M.J., Donati C., Medini D., Ward N.L., Angiuoli S.V., Crabtree J., Jones A.L., Durkin A.S. (2005). Genome Analysis of Multiple Pathogenic Isolates of Streptococcus Agalactiae: Implications for the Microbial “Pan-Genome”. Proc. Natl. Acad. Sci. USA.

[46] Page A.J., Taylor B., Delaney A.J., Soares J., Seemann T., Keane J.A., Harris S.R. (2016). SNP-Sites: Rapid Efficient Extraction of SNPs from Multi-FASTA Alignments. Microb. Genom..

[47] Stamatakis A. (2014). RAxML Version 8: A Tool for Phylogenetic Analysis and Post-Analysis of Large Phylogenies. Bioinformatics.

[48] Corander J., Marttinen P., Sirén J., Tang J. (2008). Enhanced Bayesian modelling in BAPS software for learning genetic structures of populations. BMC Bioinform..

[49] Tonkin-Hill G., Lees J.A., Bentley S.D., Frost S.D.W., Corander J. (2018). RhierBAPS: An R Implementation of the Population Clustering Algorithm HierBAPS. Wellcome Open Res..

[50] Kumar S., Stecher G., Li M., Knyaz C., Tamura K. (2018). MEGA X: Molecular Evolutionary Genetics Analysis across Computing Platforms. Mol. Biol. Evol..

[51] Jia B., Raphenya A.R., Alcock B., Waglechner N., Guo P., Tsang K.K., Lago B.A., Dave B.M., Pereira S., Sharma A.N. (2017). CARD 2017: Expansion and Model-Centric Curation of the Comprehensive Antibiotic Resistance Database. Nucleic Acids Res..

[52] Gupta S.K., Padmanabhan B.R., Diene S.M., Lopez-Rojas R., Kempf M., Landraud L., Rolain J.M. (2014). ARG-Annot, a New Bioinformatic Tool to Discover Antibiotic Resistance Genes in Bacterial Genomes. Antimicrob. Agents Chemother..

[53] Feldgarden M., Brover V., Gonzalez-Escalona N., Frye J.G., Haendiges J., Haft D.H., Hoffmann M., Pettengill J.B., Prasad A.B., Tillman G.E. (2021). AMRFinderPlus and the Reference Gene Catalog Facilitate Examination of the Genomic Links among Antimicrobial Resistance, Stress Response, and Virulence. Sci. Rep..

[54] Florensa A.F., Kaas R.S., Clausen P.T.L.C., Aytan-Aktug D., Aarestrup F.M. (2022). ResFinder—An Open Online Resource for Identification of Antimicrobial Resistance Genes in next-Generation Sequencing Data and Prediction of Phenotypes from Genotypes. Microb. Genom..

[55] Katoh K., Kuma K.I., Toh H., Miyata T. (2005). MAFFT Version 5: Improvement in Accuracy of Multiple Sequence Alignment. Nucleic Acids Res..

[56] Zankari E., Allesøe R., Joensen K.G., Cavaco L.M., Lund O., Aarestrup F.M. (2017). PointFinder: A Novel Web Tool for WGS-Based Detection of Antimicrobial Resistance Associated with Chromosomal Point Mutations in Bacterial Pathogens. J. Antimicrob. Chemother..

[57] Bandoy D.D.R., Weimer B.C. (2020). Biological Machine Learning Combined with *Campylobacter* Population Genomics Reveals Virulence Gene Allelic Variants Cause Disease. Microorganisms.

[58] Lee S., Lee D.K. (2018). What Is the Proper Way to Apply the Multiple Comparison Test?. Korean J. Anesthesiol..

[59] Purcell S., Neale B., Todd-Brown K., Thomas L., Ferreira M.A.R., Bender D., Maller J., Sklar P., De Bakker P.I.W., Daly M.J. (2007). PLINK: A Tool Set for Whole-Genome Association and Population-Based Linkage Analyses. Am. J. Hum. Genet..

[60] Ferla M.P., Pagnamenta A.T., Koukouflis L., Taylor J.C., Marsden B.D. (2022). Venus: Elucidating the Impact of Amino Acid Variants on Protein Function Beyond Structure Destabilization. J. Mol. Biol..

[61] Waterhouse A., Bertoni M., Bienert S., Studer G., Tauriello G., Gumienny R., Heer F.T., De Beer T.A.P., Rempfer C., Bordoli L. (2018). SWISS-MODEL: Homology Modelling of Protein Structures and Complexes. Nucleic Acids Res..

[62] Rodrigues C.H.M., Pires D.E.V., Ascher D.B. (2021). DynaMut2: Assessing Changes in Stability and Flexibility upon Single and Multiple Point Missense Mutations. Protein Sci..

[63] Guillier L., Gourmelon M., Lozach S., Cadel-Six S., Vignaud M.L., Munck N., Hald T., Palma F. (2020). AB_SA: Accessory Genes-Based Source Attribution—Tracing the Source of *Salmonella enterica* Typhimurium Environmental Strains. Microb. Genom..

[64] Fu S., Hiley L., Octavia S., Tanaka M.M., Sintchenko V., Lan R. (2017). Comparative Genomics of Australian and International Isolates of *Salmonella* Typhimurium: Correlation of Core Genome Evolution with CRISPR and Prophage Profiles. Sci. Rep..

[65] Petitt R.J. (1996). Measwring and Testing Genetic Differentiation With Ordered Versus Unordered Alleles. Genetics.

[66] Almeida F., Seribelli A.A., Cazentini Medeiros M.I., Rodrigues D.D.P., De MelloVarani A., Luo Y., Allard M.W., Falcão J.P. (2018). Phylogenetic and Antimicrobial Resistance Gene Analysis of *Salmonella* Typhimurium Strains Isolated in Brazil by Whole Genome Sequencing. PLoS ONE.

[67] Silva C., Betancor L., García C., Astocondor L., Hinostroza N., Bisio J., Rivera J., Perezgasga L., Escanda V.P., Yim L. (2017). Characterisation of *Salmonella enterica* Isolates Causing Bacteremia in Lima, Peru, Using Multiple Typing Methods. PLoS ONE.

[68] Quesada A., Reginatto G.A., Español A.R., Colantonio L.D., Burrone M.S. (2016). Antimicrobial Resistance of *Salmonella* spp. Isolated Animal Food for Human Consumption. Rev. Peru. Med. Exp. Salud Publica.

[69] Viana C., Grossi J.L., Sereno M.J., Yamatogi R.S., Bersot L.D.S., Call D.R., Nero L.A. (2020). Phenotypic and Genotypic Characterisation of Non-Typhoidal *Salmonella* Isolated from a Brazilian Pork Production Chain. Food Res. Int..

[70] Huamán M., Pérez C., Rodríguez J., Killerby M., Lovón S., Chauca L. (2020). Genetic Characterization and Antimicrobial Resistance Patterns of *Salmonella enterica* Subsp. Enterica Serovar Typhimurium in Guinea Pigs under Intensive Breeding. Rev. Investig. Vet. Del Perú.

[71] Guillermo S.R., Rocío R., Ana C.O., Iván R.W., Raúl R.A., Lenin M.H. (2018). Antimicrobial Resistance and Genotyping of *Salmonella* Typhimurium Strains Isolated from Guinea Pigs (*Cavia Porcellus*) from Intensive Production Farms of the City of Lima, Peru. Rev. Investig. Vet. Del Perú.

[72] Ríos C.A., Morales-Cauti S., Vilca L.M., Carhuallanqui P.A., Ramos D.D. (2019). Determinación Del Perfil de Resistencia Antibiótica de *Salmonella enterica* Aislada de Cerdos Faenados En Un Matadero de Lima, Perú. Rev. Investig. Vet. Del Perú.

[73] McDermott P.F., Tyson G.H., Kabera C., Chen Y., Li C., Folster J.P., Ayers S.L., Lam C., Tate H.P., Zhao S. (2016). Whole-Genome Sequencing for Detecting Antimicrobial Resistance in Nontyphoidal *Salmonella*. Antimicrob. Agents Chemother..

[74] Víctor Carhuapoma D., Nicasio Valencia M., Rufino Paucar C., Mayhua P.H.M., Rodrigo Huamán J., Lizana-Hilario E. (2019). Efecto de *Escherichia Coli* y *Salmonella* spp. En El Crecimiento y Mortalidad de Crías de Alpacas (*Vicugna pacos*). Rev. Investig. Vet. Del Perú.

[75] Chang M.X., Zhang J.F., Sun Y.H., Li R.S., Lin X.L., Yang L., Webber M.A., Jiang H.X. (2021). Contribution of Different Mechanisms to Ciprofloxacin Resistance in *Salmonella* spp.. Front. Microbiol..

[76] Adhikari D., Acharya D., Shrestha P., Amatya R. (2012). Ciprofloxacin Susceptibility of *Salmonella* Enteric Serovar Typhi and Paratyphi A from Blood Samples of Suspected Enteric Fever Patients. Int. J. Infect. Microbiol..

[77] Olson N.D., Lund S.P., Colman R.E., Foster J.T., Sahl J.W., Schupp J.M., Keim P., Morrow J.B., Salit M.L., Zook J.M. (2015). Best Practices for Evaluating Single Nucleotide Variant Calling Methods for Microbial Genomics. Front. Genet..

[78] Liu Z., Niu H., Wu S., Huang R. (2014). CsgD Regulatory Network in a Bacterial Trait-Altering Biofilm Formation. Emerg. Microbes Infect..

[79] Hughes D., Andersson D.I. (2017). Environmental and Genetic Modulation of the Phenotypic Expression of Antibiotic Resistance. FEMS Microbiol. Rev..

[80] Urmi U.L., Nahar S., Rana M., Sultana F., Jahan N., Hossain B., Alam M.S., Mosaddek A.S.M., McKimm J., Rahman N.A.A. (2020). Genotypic to Phenotypic Resistance Discrepancies Identified Involving β-Lactamase Genes, bla KPC, bla IMP, bla NDM-1, and bla VIM in Uropathogenic Klebsiella pneumoniae. Infect. Drug Resist..

[81] Silva C., Puente J.L., Calva E. (2017). Salmonella Virulence Plasmid: Pathogenesis and Ecology. Pathog. Dis..

[82] Tate H., Li C., Nyirabahizi E., Tyson G.H., Zhao S., Rice-Trujillo C., Jones S.B., Ayers S., M’Ikanatha N.M., Hanna S. (2021). A National Antimicrobial Resistance Monitoring System Survey of Antimicrobial-Resistant Foodborne Bacteria Isolated from Retail Veal in the United States. J. Food Prot..

[83] Matamoros S., van Hattem J.M., Arcilla M.S., Willemse N., Melles D.C., Penders J., Vinh T.N., Thi Hoa N., Bootsma M.C.J., van Genderen P.J. (2017). Global Phylogenetic Analysis of Escherichia Coli and Plasmids Carrying the Mcr-1 Gene Indicates Bacterial Diversity but Plasmid Restriction. Sci. Rep..

[84] Luo Q., Wang Y., Xiao Y. (2020). Prevalence and Transmission of Mobilized Colistin Resistance (Mcr) Gene in Bacteria Common to Animals and Humans. Biosaf. Health.

[85] Raz R. (2012). Fosfomycin: An Old—New Antibiotic. Clin. Microbiol. Infect..

[86] Liu Q., Chen W., Elbediwi M., Pan H., Wang L., Zhou C., Zhao B., Xu X., Li D., Yan X. (2020). Characterisation of *Salmonella* Resistome and Plasmidome in Pork Production System in Jiangsu, China. Front. Vet. Sci..

[87] Güneri C.Ö., Stingl K., Grobbel M., Hammerl J.A., Kürekci C. (2022). Different FosA Genes Were Found on Mobile Genetic Elements in Escherichia Coli from Wastewaters of Hospitals and Municipals in Turkey. Sci. Total Environ..

[88] Wong V.K., Baker S., Pickard D.J., Parkhill J., Page A.J., Feasey N.A., Kingsley R.A., Thomson N.R., Keane J.A., Weill F.X. (2015). Phylogeographical Analysis of the Dominant Multidrug-Resistant H58 Clade of *Salmonella* Typhi Identifies Inter-and Intracontinental Transmission Events. Nat. Genet..

[89] Chen W., Fang T., Zhou X., Zhang D., Shi X., Shi C. (2016). IncHI2 Plasmids Are Predominant in Antibiotic-Resistant *Salmonella* Isolates. Front. Microbiol..

[90] Li L., Liao X., Yang Y., Sun J., Li L., Liu B., Yang S., Ma J., Li X., Zhang Q. (2013). Spread of OqxAB in *Salmonella enterica* Serotype Typhimurium Predominantly by IncHI2 Plasmids. J. Antimicrob. Chemother..

[91] Ministerio de Salud (Minsa) (2017). Plan Nacional Para Enfrentar La Resistencia a Los Antimicrobianos 2017–2021. Perú. https://www.digemid.minsa.gob.pe/UpLoad/UpLoaded/PDF/Acceso/URM/GestionURMTrabSalud/ReunionTecnica/VIII/Dia2/Antimicrobianos/PlanNacionalATM-2017-2021.pdf.

[92] Ashley R.E., Dittmore A., McPherson S.A., Turnbough C.L., Neuman K.C., Osheroff N. (2017). Activities of Gyrase and Topoisomerase IV on Positively Supercoiled DNA. Nucleic Acids Res..

[93] Shaheen A., Tariq A., Iqbal M., Mirza O., Haque A., Walz T., Rahman M. (2021). Mutational Diversity in the Quinolone Resistance-Determining Regions of Type-II Topoisomerases of *Salmonella* Serovars. Antibiotics.

[94] Li X.Z., Plésiat P., Nikaido H. (2015). The Challenge of Efflux-Mediated Antibiotic Resistance in Gram-Negative Bacteria. Clin. Microbiol. Rev..

[95] Rahmati S., Yang S., Davidson A.L., Zechiedrich E.L. (2002). Control of the AcrAB Multidrug Efflux Pump by Quorum-Sensing Regulator SdiA. Mol. Microbiol..

[96] Martínez-Puchol S., Pons M.J., Ruiz-Roldán L., Laureano-Adame L., Corujo A., Ochoa T.J., Ruiz J. (2020). Nitrofuran Resistance in *Salmonella enterica* Isolated from Meat for Human Consumption. Rev. Peru. Med. Exp. Salud Publica.

[97] Miryala S.K., Ramaiah S. (2019). Exploring the Multi-Drug Resistance in Escherichia Coli O157:H7 by Gene Interaction Network: A Systems Biology Approach. Genomics.

[98] Zhang Y.E., Bærentsen R.L., Fuhrer T., Sauer U., Gerdes K., Brodersen D.E. (2019). (P)PpGpp Regulates a Bacterial Nucleosidase by an Allosteric Two-Domain Switch. Mol. Cell.

[99] Lin S., Hanson R.E., Cronan J.E. (2010). Biotin Synthesis Begins by Hijacking the Fatty Acid Synthetic Pathway. Nat. Chem. Biol..

[100] Al-Dabbagh B., Mengin-Lecreulx D., Bouhss A. (2008). Purification and Characterization of the Bacterial UDP-GlcNAc:Undecaprenyl-Phosphate GlcNAc-1-Phosphate Transferase WecA. J. Bacteriol..

