# Supplementary material for: WGS-Based Lineage and Antimicrobial Resistance Pattern of Salmonella Typhimurium Isolated during 2000–2017 in Peru
Source: Antibiotics (Basel). 2022 Aug 30;11(9):1170. doi: 10.3390/antibiotics11091170 (PMC9495214; doi:10.3390/antibiotics11091170)

Figure S1. Whole-genome Average Nucleotide Identity (ANI) and sequence type: A) 140 peruvian and global *Salmonella* Typhimurium strains B) 90 peruvian *Salmonella* Typhimurium strains.

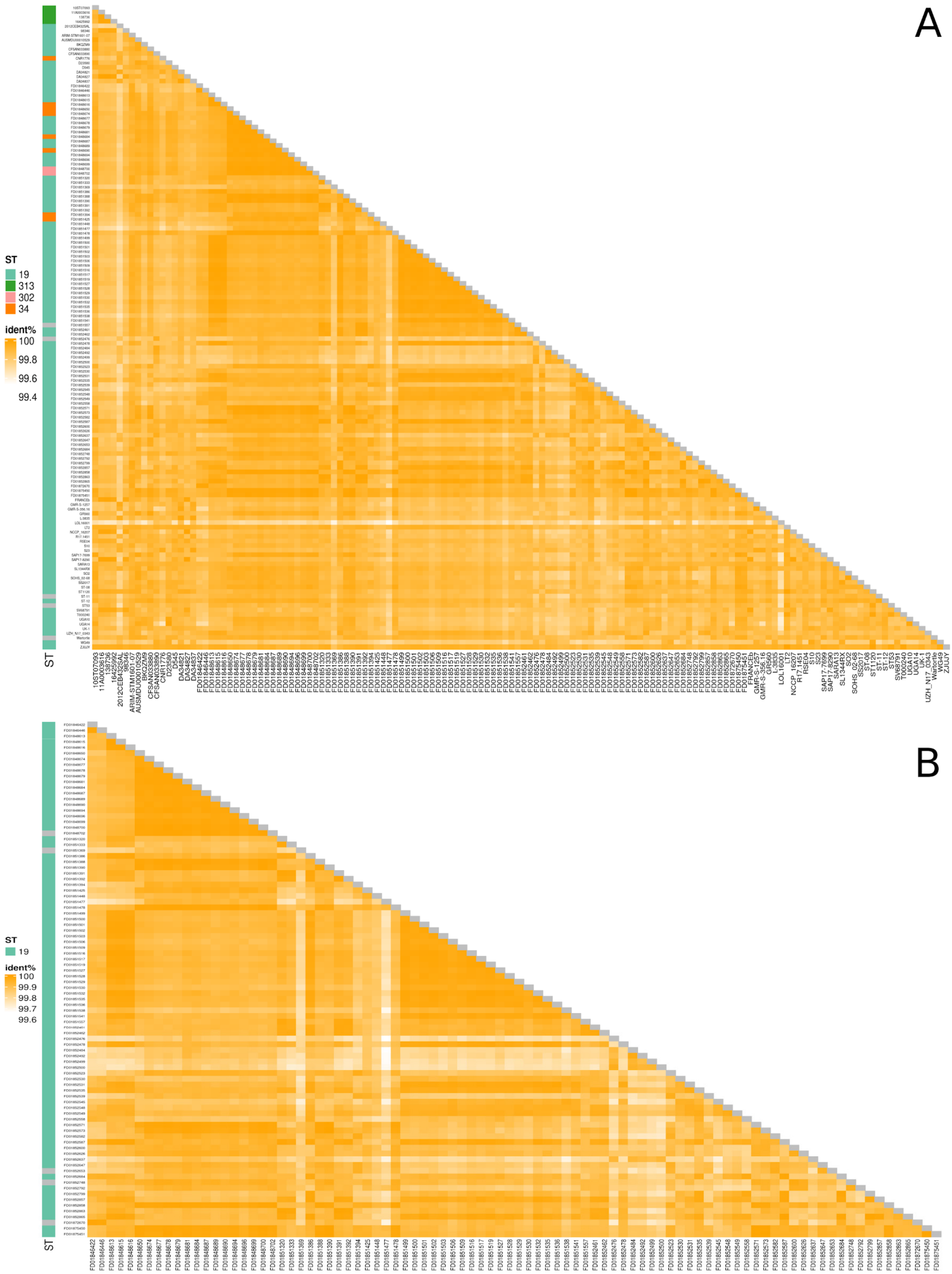

Figure S2. Nucleotide similarity and sequence type: A) 140 peruvian and global *Salmonella* Typhimurium strains B) 90 peruvian *Salmonella* Typhimurium strains.

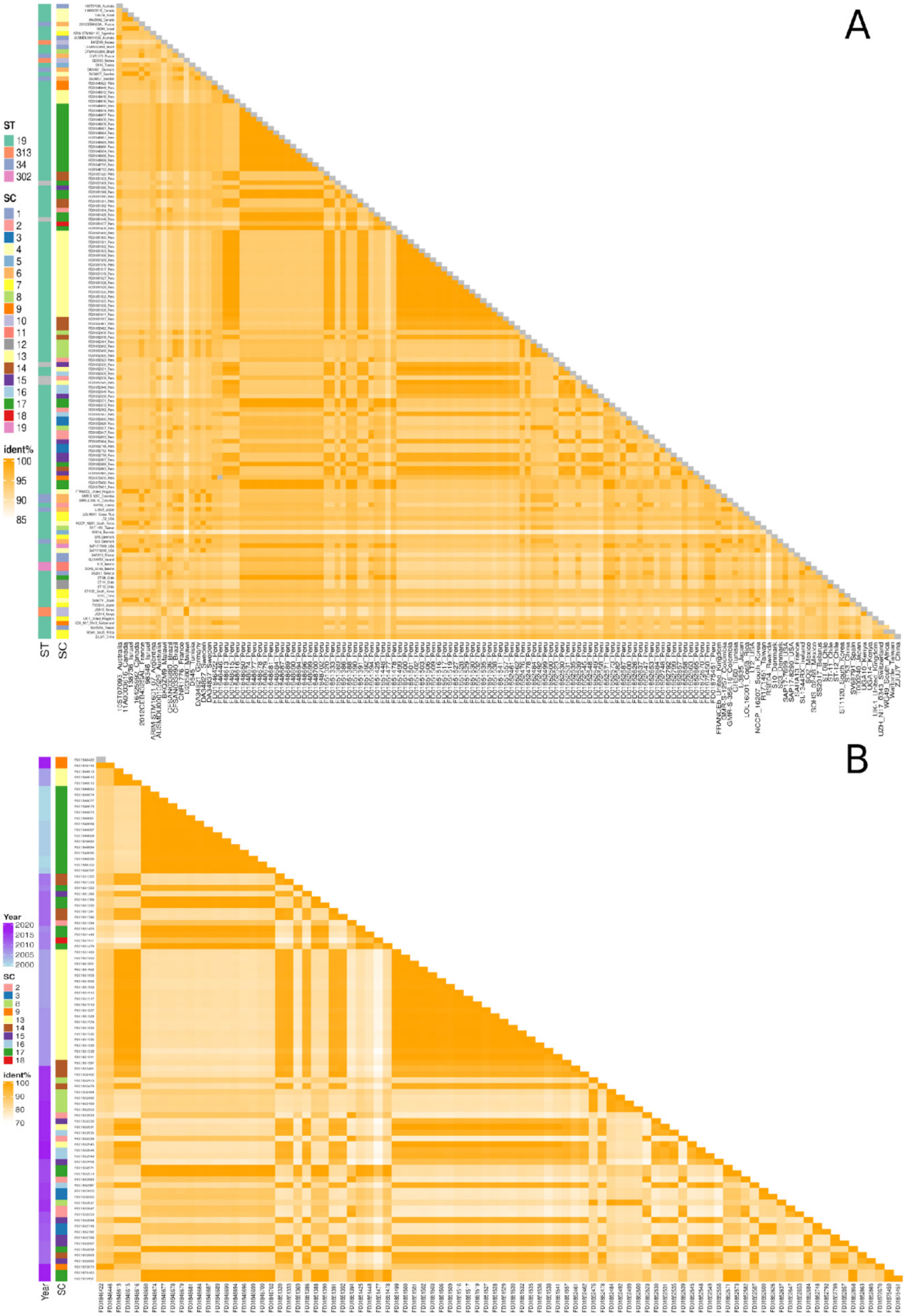

Figure S3. Phylogenetic tree was generated using a maximum likelihood method with 1000 replicates of bootstrap using GTR+GAMMA to estimate evolutionary distance between 140 global isolates. The phylogenetic tree was clustered into at least two large clades and segregated into at least nineteen sub-clades.

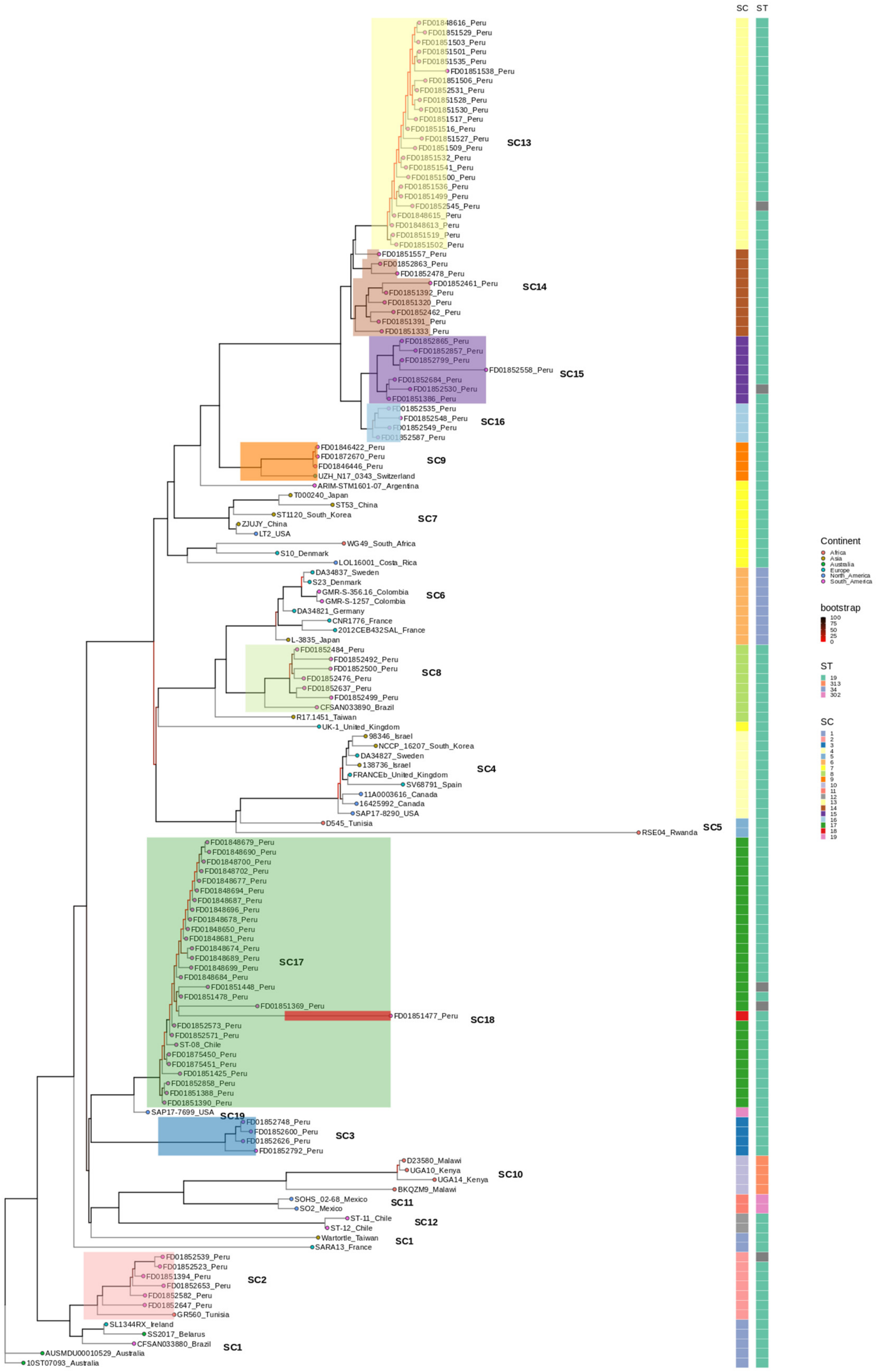

Supplement: Supplementary file 1 [file antibiotics-11-01170-s001.zip › Supplementary Figures S1-S3.pdf]
