# Supplementary material for: WGS-Based Lineage and Antimicrobial Resistance Pattern of Salmonella Typhimurium Isolated during 2000–2017 in Peru
Source: Antibiotics (Basel). 2022 Aug 30;11(9):1170. doi: 10.3390/antibiotics11091170 (PMC9495214; doi:10.3390/antibiotics11091170)
Supplement: Supplementary file 1 [file antibiotics-11-01170-s001.zip › Supplementary material S1.pdf]

**Supplementary material S1.** Model quality validation: The 3D structures were generated using SWISS-MODEL and evaluated using Ramachandram plot.

## Analysis of structure proteins with to new and known variants on AMR genes

### ***parC***

Swiss Model template- PDB: 1zvu.1.A, identity is 71%.

Selected model: 1, chain A

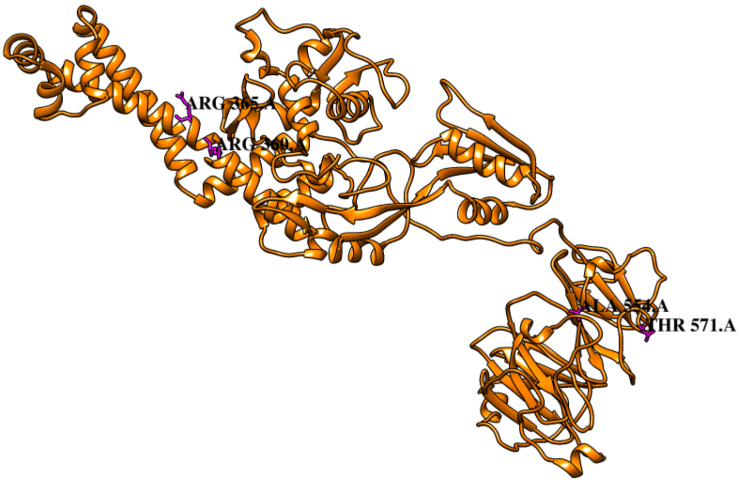

**Ramachandran Plot result:**

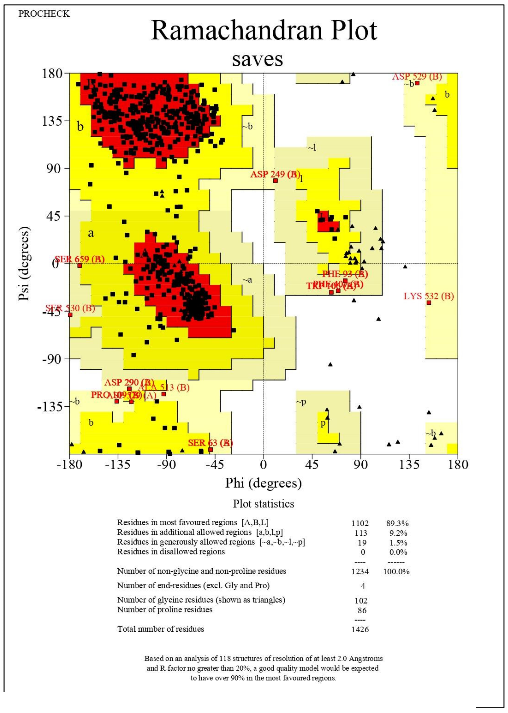


***gyrA***

**Swiss Model template-PDB**: 6rkv.1.C, identity is 67%,

Selected model no: 2, chain B

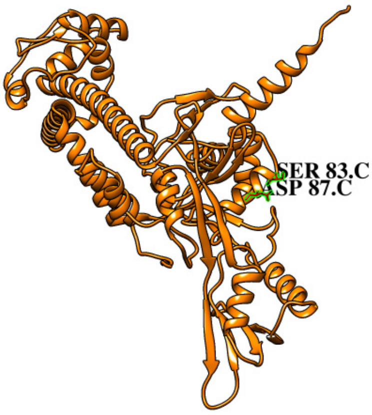


**Ramachandran Plot result:**

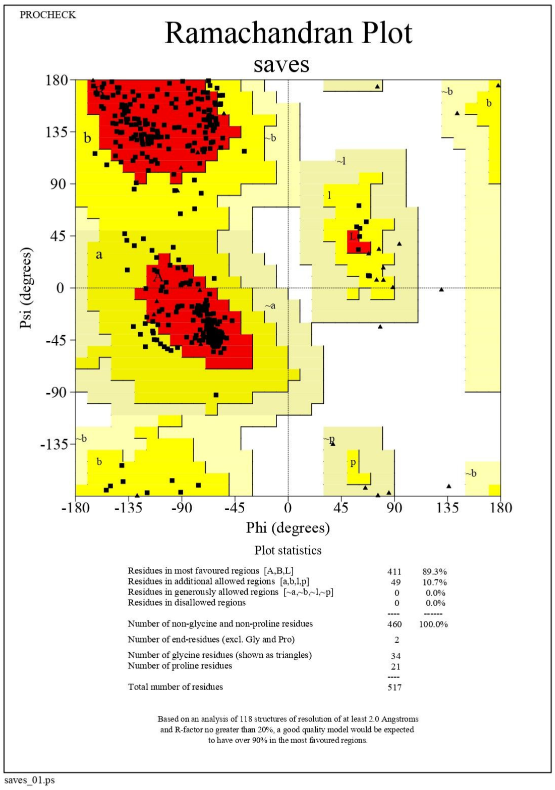


***<u>sdiA</u>***

Swiss Model template- PDB: 4y15.2.A, identity is 82%.

Selected model no: 1, chain A

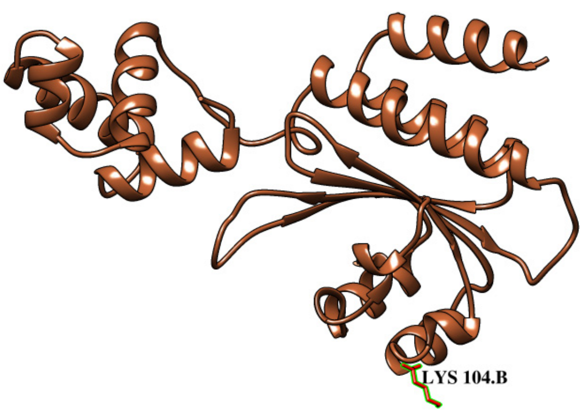

**Ramachandran Plot result:**

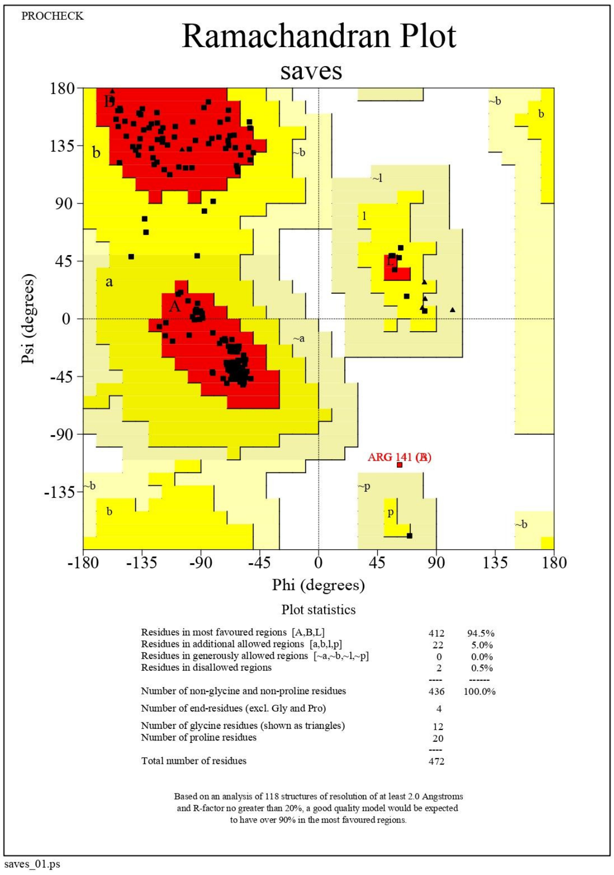


***nfsa***

Swiss Model template-PDB: 1f5v.1.A, identity is 92%.

Selected model no: 1

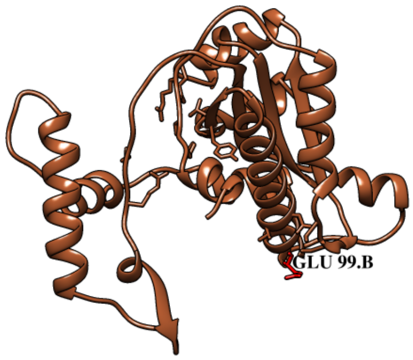

**Ramachandran Plot result:**

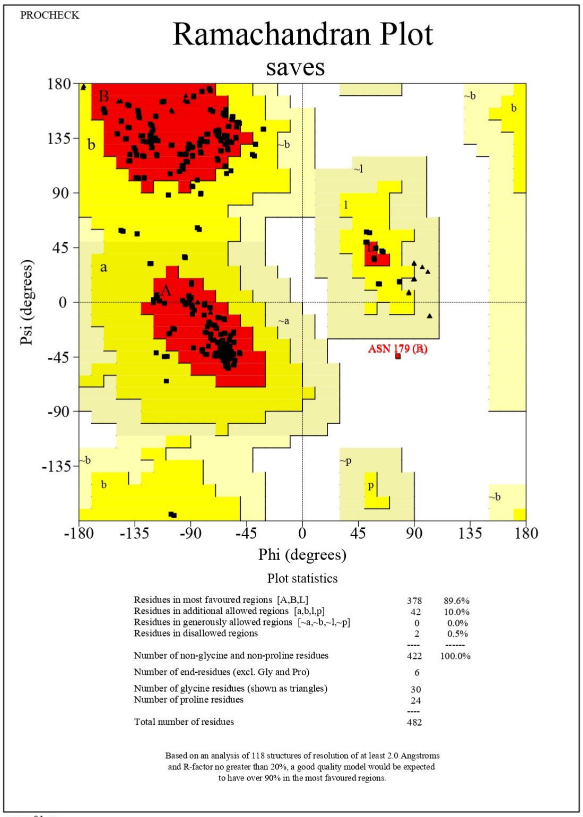

saves_01.ps

## Analysis of structure proteins with GWAS-based new variants.

***ppnn***

Swiss Model template- PDB:2pmb.1.B, identity is 68%.

Selected model no: 1, chain A

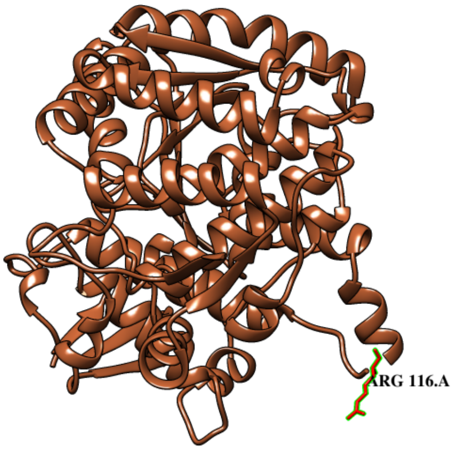


**Ramachandran Plot result:**

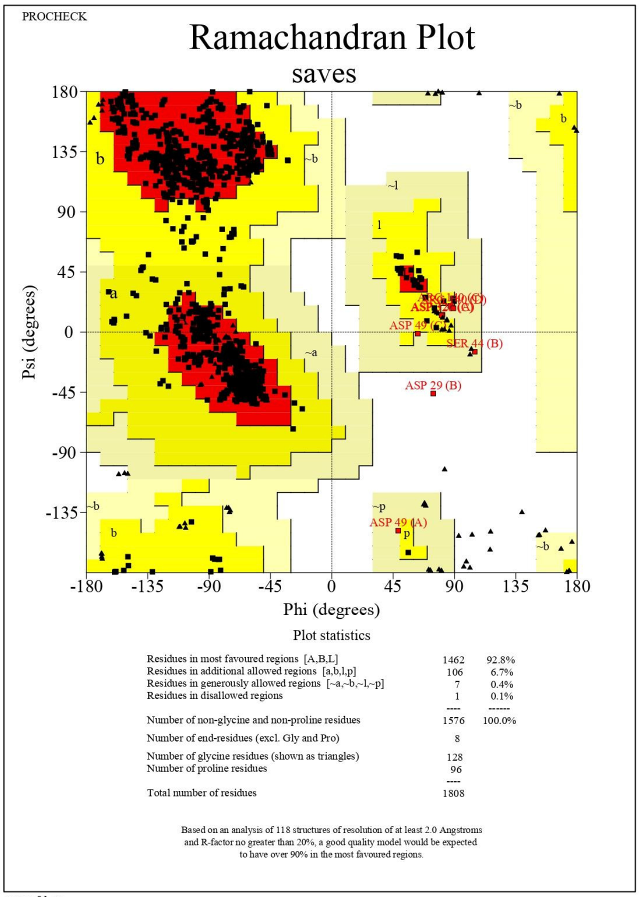


saves_01.ps

***bioH***

Swiss Model template-PDB:4nmw.1.A, identity is 100%

Selected model no: 1, chain A

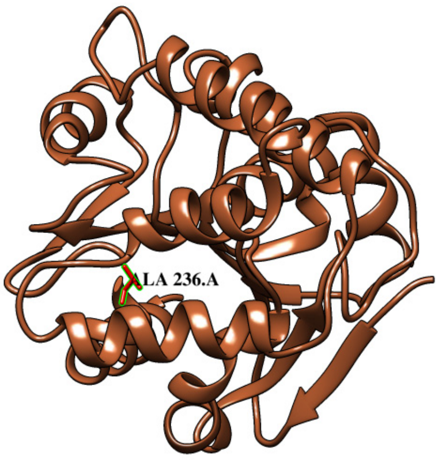


**Ramachandran Plot result:**

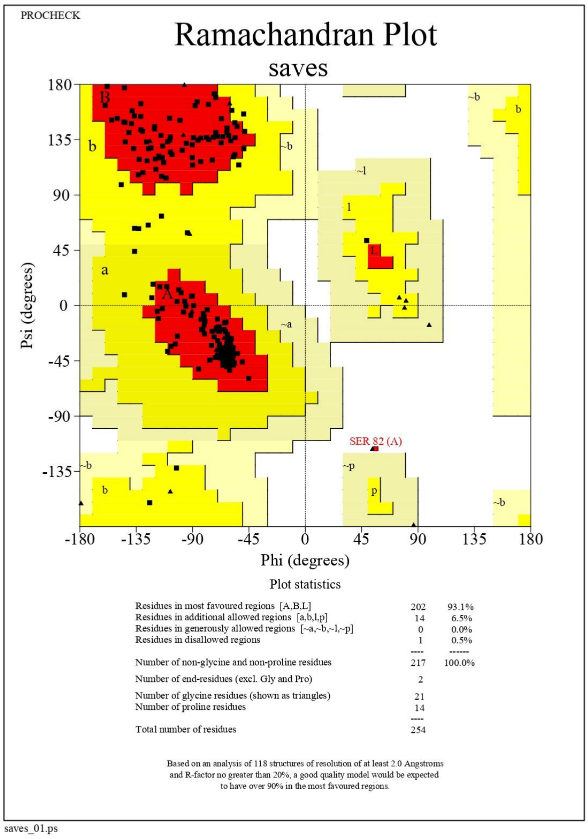


***mdtH***

Swiss-model- PDB: 3wdo.1.A, identity is 20.06%.

Selected model no: 1

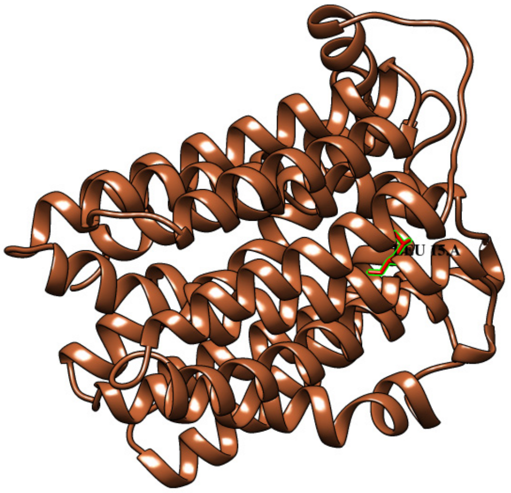

**Ramachandran Plot result:**

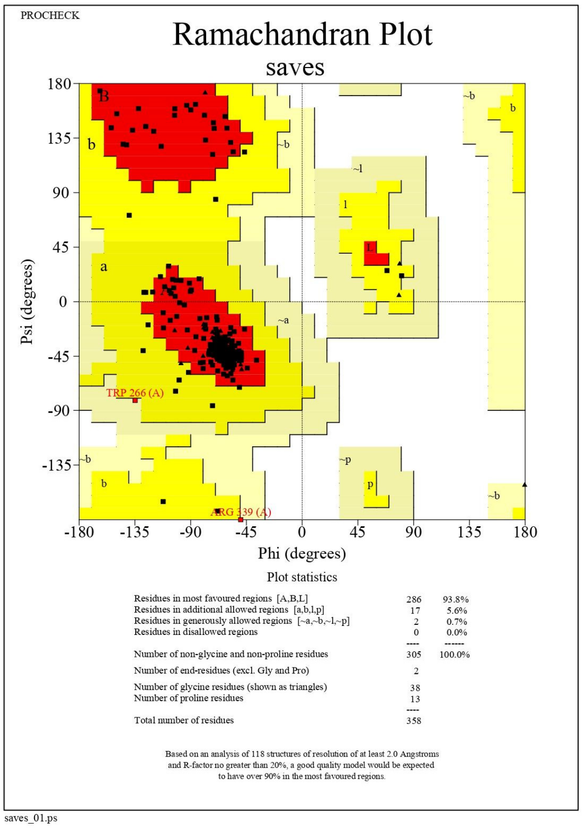


## qseC

Template- PDB: 2kse.1.A, identity is 71.43%.

Selected model no: 3

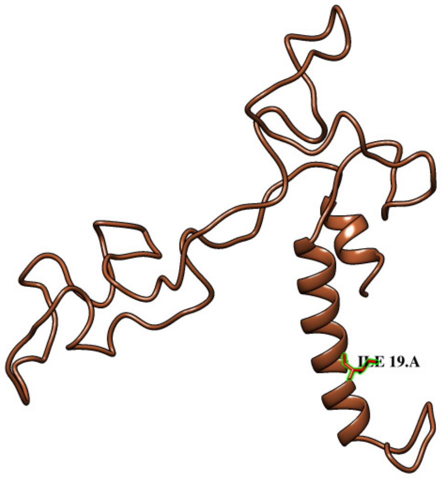


**Ramachandran Plot result**:

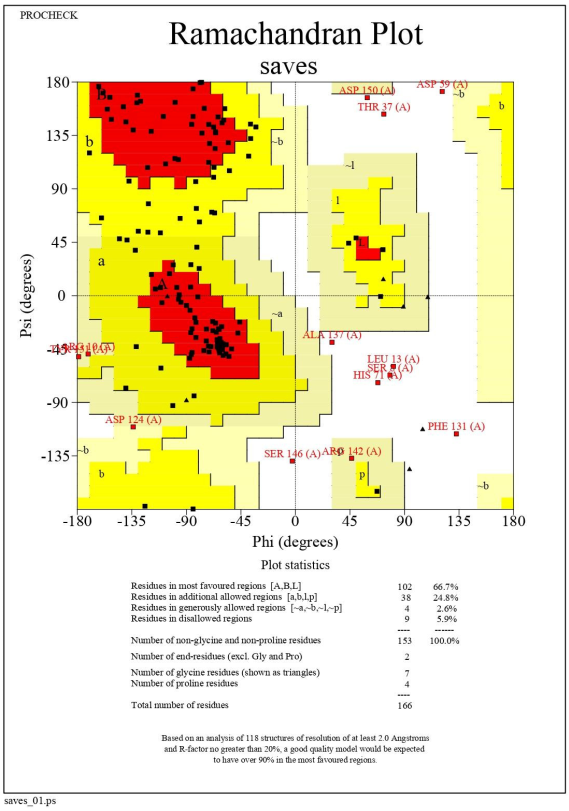


***purA***

Template- PDB: 2gcq.1.A , identity is 100%.

Selected model no: 1

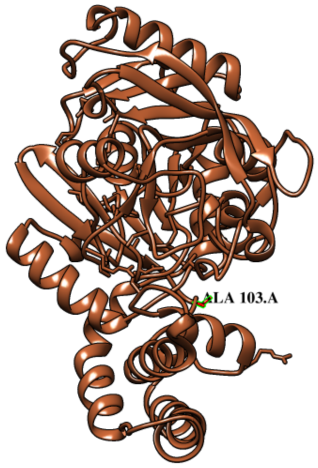


**Ramachandran Plot result:**

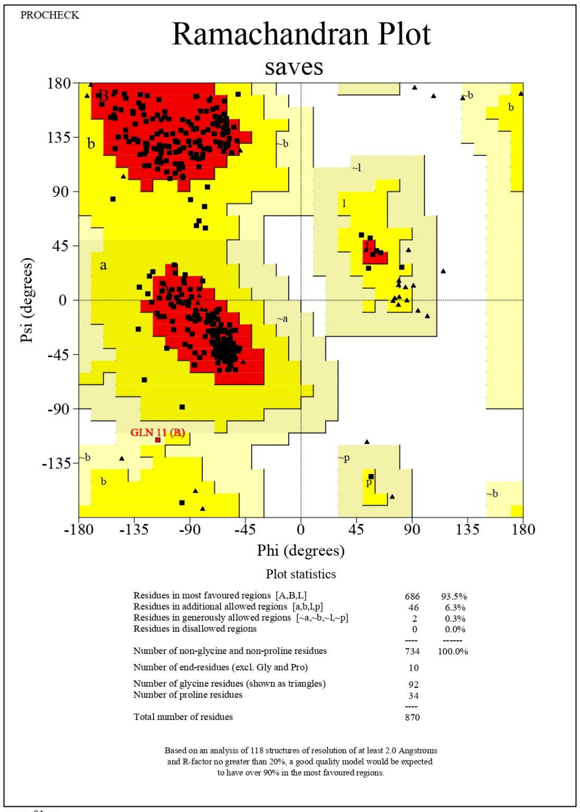

saves_01.ps

***wecA***

Template-identity is 33% PDB:5jnq.1.A,

Selected model no: 1

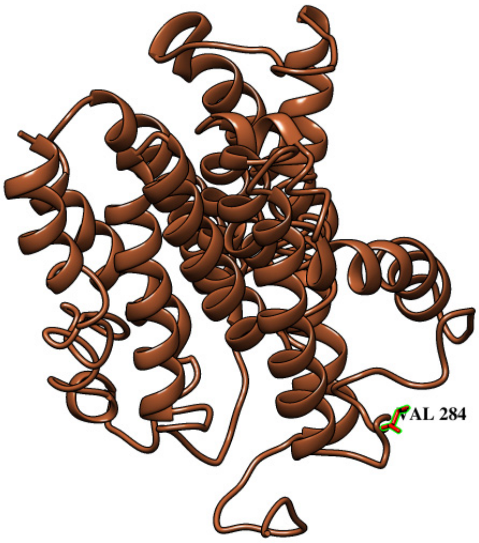


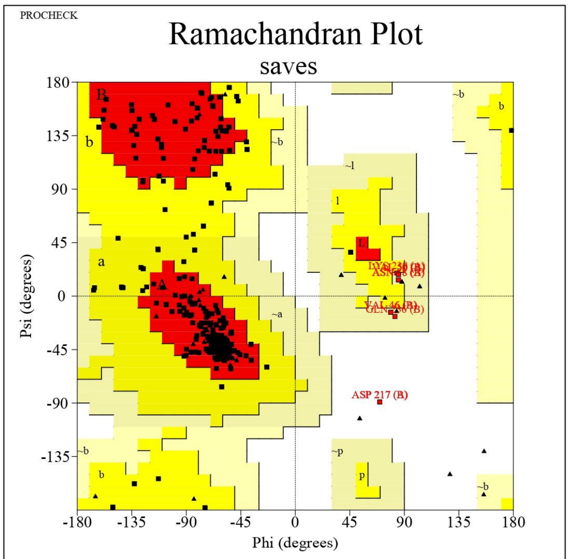


Plot statistics

| | | |
|---|---|---|
| Residues in most favoured regions [A,B,L] | 475 | 88.3% |
| Residues in additional allowed regions [a,b,l,p] | 53 | 9.9% |
| Residues in generously allowed regions [~a,~b,~l,~p] | 8 | 1.5% |
| Residues in disallowed regions | 2 | 0.4% |
| | ---- | ------ |
| Number of non-glycine and non-proline residues | 538 | 100.0% |
| Number of end-residues (excl. Gly and Pro) | 4 | |
| Number of glycine residues (shown as triangles) | 58 | |
| Number of proline residues | 20 | |
| | ---- | |
| Total number of residues | 620 | |

Based on an analysis of 118 structures of resolution of at least 2.0 Angstroms and R-factor no greater than 20%, a good quality model would be expected to have over 90% in the most favoured regions.

saves_01.ps
